# Cortical and Hippocampal Correlates of Deliberation during Model-Based Decisions for Rewards in Humans

**DOI:** 10.1371/journal.pcbi.1003387

**Published:** 2013-12-05

**Authors:** Aaron M. Bornstein, Nathaniel D. Daw

**Affiliations:** 1 Department of Psychology, Program in Cognition and Perception, New York University, New York, New York, United States of America; 2 Center for Neural Science, New York University, New York, New York, United States of America; University of Oxford, United Kingdom

## Abstract

How do we use our memories of the past to guide decisions we've never had to make before? Although extensive work describes how the brain learns to repeat rewarded actions, decisions can also be influenced by associations between stimuli or events not directly involving reward — such as when planning routes using a cognitive map or chess moves using predicted countermoves — and these sorts of associations are critical when deciding among novel options. This process is known as model-based decision making. While the learning of environmental relations that might support model-based decisions is well studied, and separately this sort of information has been inferred to impact decisions, there is little evidence concerning the full cycle by which such associations are acquired and drive choices. Of particular interest is whether decisions are directly supported by the same mnemonic systems characterized for relational learning more generally, or instead rely on other, specialized representations. Here, building on our previous work, which isolated dual representations underlying sequential predictive learning, we directly demonstrate that one such representation, encoded by the hippocampal memory system and adjacent cortical structures, supports goal-directed decisions. Using interleaved learning and decision tasks, we monitor predictive learning directly and also trace its influence on decisions for reward. We quantitatively compare the learning processes underlying multiple behavioral and fMRI observables using computational model fits. Across both tasks, a quantitatively consistent learning process explains reaction times, choices, and both expectation- and surprise-related neural activity. The same hippocampal and ventral stream regions engaged in anticipating stimuli during learning are also engaged in proportion to the difficulty of decisions. These results support a role for predictive associations learned by the hippocampal memory system to be recalled during choice formation.

## Author Summary

We are always learning regularities in the world around us: where things are, and in what order we might find them. Our knowledge of these contingencies can be relied upon if we later want to use them to make decisions. However, there is little agreement about the neurobiological mechanism by which learned contingencies are deployed for decision making. These are different kinds of decisions than simple habits, in which we take actions that have in the past given us reward. Neural mechanisms of habitual decisions are well-described by computational reinforcement learning approaches, but have not often been applied to ‘model-based’ decisions that depend on learned contingencies. In this article, we apply reinforcement learning to investigate model-based decisions. We tested participants on a serial reaction time task with changing sequential contingencies, and choice probes that depend on these contingencies. Fitting computational models to reaction times, we show that two sets of predictions drive simple response behavior, only one of which is used to make choices. Using fMRI, we observed learning and decision-related activity in hippocampal and ventral cortical areas that is computationally linked to the learned contingencies used to make choices. These results suggest a critical role for a hippocampal-cortical network in model-based decisions for reward.

## Introduction

Every day, we learn new information that is not immediately relevant to our current goals. We might learn the layout of a new neighborhood, or, while traveling a familiar street, happen upon a restaurant that is about to open. Though we might not receive any rewards — e.g., a friendly neighbor or a great meal — during our initial experience, we still learn our way around. If, later, we decide to seek a particular reward, we are usually quite capable of using the knowledge we gained from such exploration to achieve our goal. This is known as goal-directed or model-based decision making: the construction of plans to achieve rewards, incorporating knowledge about contingencies in the world [1]–[3]. The neural systems that support these forms of decisions are a focus of much ongoing research.

In this study, we provide evidence that the hippocampus and related cortical regions support the contingencies necessary to perform model-based decisions. We show that ongoing learning of the required contingencies can be measured in two kinds of behavior: simple responses and deliberative choices. Further, we show that BOLD signal in the regions of interest scales with multiple computational variables that describe the use of these contingencies to perform action selection.

### Representations in model-based decisions

Model-based decisions stand in contrast to a simpler sort of learned decision making whose neural instantiation is better understood: simply learning to repeat rewarded behaviors [4]–[6]. To explain the former, more knowledge-driven path to decisions, researchers have long argued that the brain maintains internal representations of the contingency structure of a task — a “world model” or, in spatial tasks, a “cognitive map” — that can be adaptively applied to drive behavior. Like a map of space, these representations describe the relationships between situations and actions, separate from any ties to reward. The reliance on these representations is a defining characteristic of goal-directed decisions [1], [2]. Therefore, to identify the neural mechanisms of these decisions, researchers must first identify the representations that guide them.

### From learning to action

Here, to examine in detail the process by which contingency representations are learned and inform action choice, we combined a sequential learning task [7] with an interleaved decision task in which rewards depended on contingencies learned in the first task. In the learning task, participants were presented with one of four photograph images at a time, and asked simply to press the key corresponding to that image. Which of the four images appeared next depended, probabilistically, on the image currently being viewed. The sequential learning task allowed us to measure the gradual, trial-by-trial, acquisition of these probabilistic contingencies linking the four image stimuli. Participants' responses provided two observable measurements of learning: reaction time to identify each image, and image-specific BOLD activity in the ventral stream visual cortex.

Reaction times to identify an image indicated the degree to which subjects expected it, given the previous one — a classic and relatively direct measure of the learned predictive association [8]–[12] — and category-specific BOLD also reflected engagement of the neural representation of each image in anticipation of its presentation [13]. By fitting computational models to this progression of subject expectations, we extracted a computational signature of the learning process, the *learning rate*, and used it to generate timeseries of decision variables based on these learned contingencies.

This enabled us to quantitatively characterize the influence of these associations when participants were asked, in the interleaved decision probes, to draw on them to make decisions. Specifically, participants were told that one of the four images was, for a short period of time, to be associated with a reward. They were then asked which of two other images would lead to that rewarded image as quickly as possible. This manipulation has a form similar to a latent learning paradigm [14], [15], in which contingencies are learned separately from their link to reward. By requiring subjects to use knowledge of the contingencies to guide their decisions, this design allows us to probe how and whether the contingencies are used to seek trial-specific goals — contingencies that are exclusively the realm of model-based decision processes.

Comparing the learning rates fit to behavior and BOLD responses we observed a striking match between hippocampal correlates of sequential learning and the learning underlying the reaction times, choices, prediction errors, and ventral visual stream activity, during both simple identification responses and deliberative decisions for reward. These results suggest that regions involved in sequential learning, including hippocampus and ventral cortical areas, indeed provide the necessary contingency representations to support model-based choice — and, critically, demonstrate the use of particular associations learned by these regions during model-based decision making.

## Results

**Figure 1:**
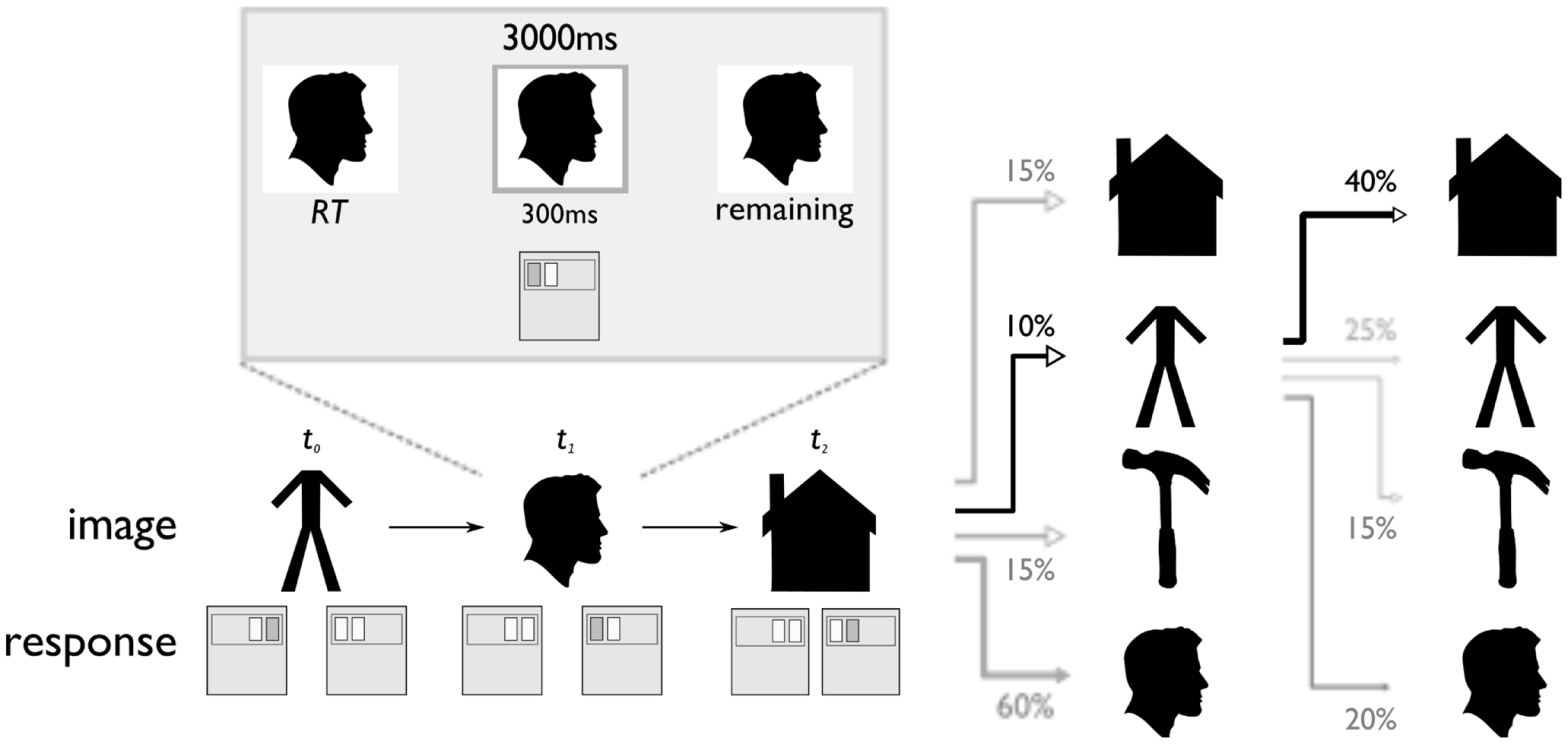
Serial reaction time task. Images were presented one at a time for a fixed 3000-order Markov transition process (i.e., a 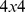 matrix of conditional probabilties). The conditional probabilities were changed abruptly at three points during the task, unaligned to rest periods and with no visual or other notification. (Images shown here are not those used in the study, but public domain stand-ins from clker.com that reflect the category of the photographs used during the experiment.)

**Figure 2:**
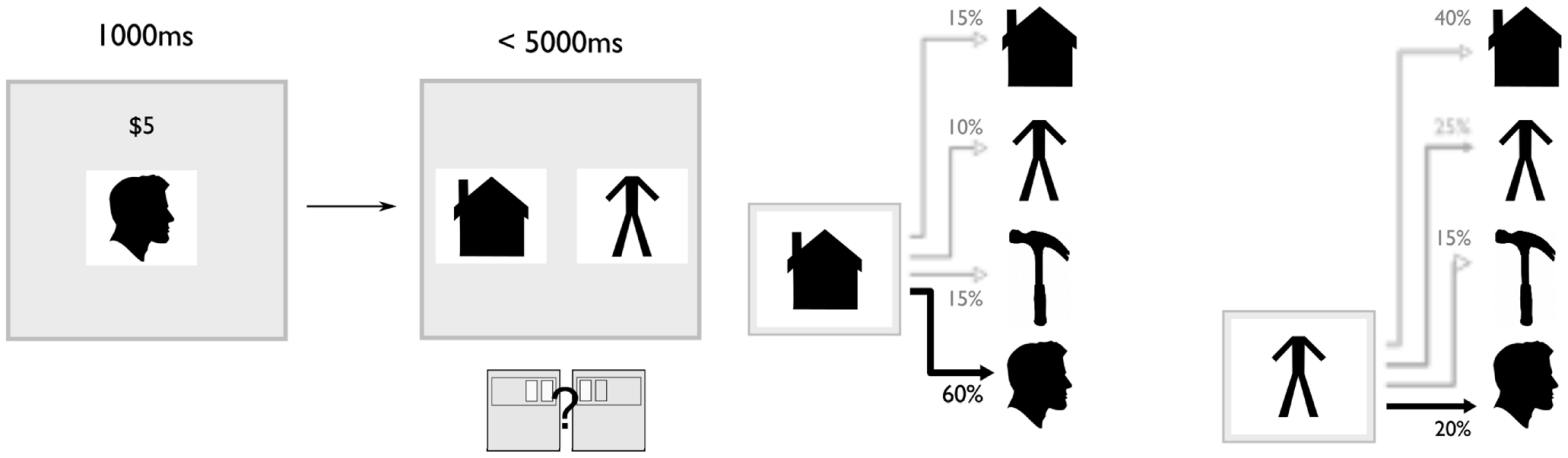
Choice task. Participants were asked to use their knowledge of the sequential transition structure to make decisions for reward. Choice rounds consisted of three steps. First, participants observed the reward amount and target image for one second. Next, they were given five seconds to choose one of two images to start the sequence from again. This choice was of varying difficulty, depending on how likely it was for each choice image to be followed by the reward image. For the next several presentations after choice, each observation of the valued image was accompanied by reward. (Images shown here are not those used in the study, but public domain stand-ins from clker.com that reflect the category of the photographs used during the experiment.)

Our task trains participants on probabilistic sequential contingencies linking image stimuli (Figure 1). Then, on probe trials interspersed with the learning, the task offers participants the opportunity to make decisions for rewards, using their estimates of those sequential contingencies to inform their choices (Figure 2). Previously, we showed that two neural processes — associated with the hippocampus and striatum, respectively — develop separate estimates of the contingencies in the learning portion of this task [7]. As the hippocampal system has long been a candidate for learning the relations (e.g., maps or models) supporting flexible choice, our hypothesis is that goal-directed decisions will depend on the contingency estimates learned by the hippocampal system.

To test this hypothesis, we fit computational learning models to explain behavioral and neural observables (such as reaction times, decisions, and BOLD activity) in terms of recent experience with image transitions. Following the approach developed previously [7], for each observable we estimate a learning rate parameter, which measures how far into the past its behavior is affected by previous events. Since the learning rate measures which particular events the observable is sensitive to, we use it as signature of the underlying associative learning process. We then compare these estimates across different observables to investigate whether they might be driven by common learned associations.

We first examine reaction times for behavioral evidence of prediction learning during the sequential image presentations, verifying that the key results from the earlier study are replicated in the present design. Next, we examine how this learning is used to guide goal-directed choices for reward.

We then carry these analyses over to neuroimaging data, observing neural correlates of learned predictions across both task phases. One source of such correlates is image category-specific BOLD signals in visual ventral stream regions during the sequential learning task. During choice probes, we identify analogous content-specific activations that reflect deliberative computations supporting model-based decisions.

### Behavior

#### Two processes learn serial order relationships

Participants performed a sequential response task in which they were asked to press a key corresponding to one of four exemplar images, each displayed one at a time (Figure 1). The sequence was generated according to a first-order Markov process: at each step, an image's successor was chosen from a probability distribution over the four images. The distributions over next images were different for each current image. Participants were instructed as to the existence, but not the content, of this transition structure. They were told that these contingencies would change periodically, and without notice, throughout the experiment.

**Figure 3:**
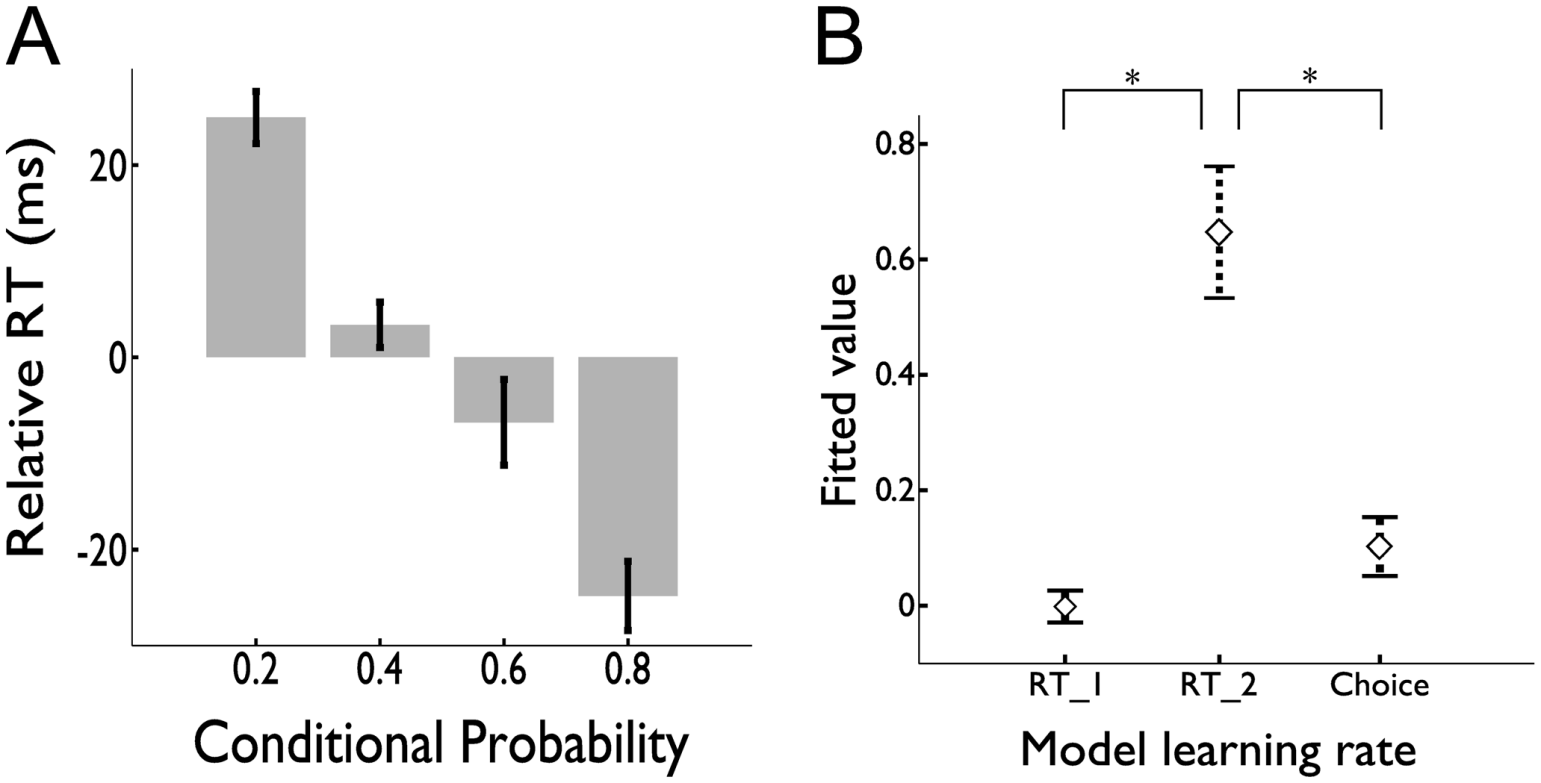
Behavioral analyses. *a*. Reaction time on the image identification task decreases as the ‘ground-truth’ probability – the probabilities generated by the task program, and uninstructed to the participant – of that image appearing, conditional on the previous image increases. Here, for each participant, RTs were first corrected for their mean and a number of nuisance effects, estimated using a linear regression containing only these effects as explanatory variables. *b*. Across subjects, the fitted learning rate values that best explain behavior. For reaction times, the best-fitting model contained two learning rates (one ‘slow’, the other ‘fast’), whose estimates were combined linearly according to a fitted weighting parameter. For choice behavior, the best-fitting model contained one learning rate, statistically indistinguishable from the slow rate fit to reaction times, but significantly different from the fast.

As has often been observed in such tasks [8], reaction times (RTs) were facilitated for images that were conditionally more probable given their predecessor (Figure 3). The impression that RTs are faster for conditionally more probable images is confirmed by performing a multiple linear regression with the ground-truth (programmed) conditional probability as the explanatory variable of interest. Across participants, the regression weight for this quantity was indeed significantly negative (one-sample t-test, 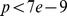; mean effect size 0.44 ms RT per percentage conditional probability) and, at an individual level, reached significance (at 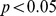) for all 17 participants.

This speeding allowed us to use RT as a behavioral index of participants' image expectation, and to leverage this to study how subjects updated their expectations trial-by-trial, by fitting computational learning models to the RT timeseries. As in our previous study [7], RTs were well explained by combining two incremental learning processes [16], [17]. The processes each separately learn a table of conditional image succession probabilities, updating it incrementally in response to the prediction error at each observation, but with the size of this update in each of the independent processes controlled by a different learning rate parameter (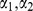). To explain reaction times, the two conditional probability predictions are combined in a weighted average with some proportion 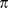. This two-process learning model provided a better fit to RTs than a one-process model for all 17 subjects individually (average log Bayes Factor 12.53, with no individual Bayes Factor in favor of the one-process model), and for the population as a whole (summed log Bayes Factor 213.08). The means, over the population, of the model's best fitting parameters were 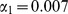, 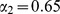, with a weight of 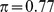 to the slower rate. To generate regressors for fMRI we refit the group's behavior, taking all parameters as fixed effects across the population. (This regularizes the parameter estimates and allows us to examine variations in neurally implied learning rate estimates relative to a common baseline.) The fixed-effect parameter estimates were 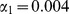 and 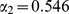, weighted at 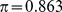, which did not significantly differ from the ensemble of individual estimates (all 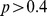).

These data are consistent with our hypothesis that sequential learning arises from two distinct learning processes, which are superimposed to produce reaction time behavior.

#### Only slow-process associations drive choice

Our next aim was to examine how these predictions were used to make decisions for reward, and in particular to what extent decisions draw on either or both of the learning processes that drive reaction times.

At pseudorandom intervals throughout the task, participants encountered a choice probe (Figure 2) in which they were asked to use their current estimates of image contingencies to make decisions for reward.

Participants were informed that one of the four images was now worth money ($1 to $5) each time it occurred during the next several trials. They were next asked to choose from which of two other images to restart the sequence, so as to maximize their chance of winning money.

To examine how learned sequential transition probabilities influence choice behavior, we fit choices with a model in which participants chose between the two starting images on the basis of the estimated probability of each image leading to the rewarded image in one step. (We did not find evidence that participants took into account the possibility that choosing an image would lead to the rewarded image on timesteps following the first.) In particular, the model assumes that the chance of choosing an option depends on a decision variable defined as the difference between the conditional probability that the rewarded image would follow each of the two options. In this model, choice preferences depend on the transition probabilities learned in the preceding sequential response trials, and therefore they also depend on the learning rate. Because each learning rate implies a different series of transition probabilities, they also imply a different timeseries of choice preferences.

We fit learning models to the choices to answer the question: Which learning rate (or rates) for transition probabilities provided the best explanation for choice behavior? Considering the possibility that, like RTs, choices were due to some weighted combination of probabilities learned at two rates, we compared one- and two-process models. However, in this case a model with a single free learning rate provided a better fit for all 17 subjects individually (mean log Bayes Factor 2.31), and across the population (summed log Bayes Factor 39.26 versus the two rate model).

This single free learning rate, fit to choices, matched the slow learning rate fit to reaction times. Across subjects, the mean best-fit learning rate was 0.10+/−0.05, which was smaller than the fast learning rate obtained for RTs (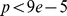) but not significantly different from the slow learning rate (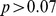) (Figure 3). These results suggest that choices, unlike reaction times, exclusively result from associations learned at a single timescale, consistent with the slow process observed in RTs.

How are these learned transition probabilities used to compute action values? The standard model is that expected values are computed by multiplying the probability of each option image leading to the goal image by the reward value of that goal image. These expected values are then transformed into choice probabilities using a softmax function, with a free parameter 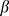.

Another approach, inspired by race models [18], is based on the idea that the outcome predictions driving choice might involve discrete retrievals of next-step images, proportional to the estimated transition probabilities [19], [20]. In this model, choice probabilities result from a thresholded comparison process after some number of draws from the binomial distribution (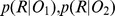) defined by the transition probabilities. This approach is similar to the sort of sequential sampling processes used to model perceptual decisions [21]. Fitting this model to the set of choices by each participant gives an additional parameter, 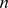, the average number of draws. Here, binomial sampling noise introduces stochasticity in the choices similar to the softmax logistic distribution often used in decision models [22], with 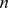 playing a role analogous to softmax's inverse temperature. (See *Materials and Methods*, section *Choice models*, for more details.) In fact, choices are also similarly fit by the softmax, and the foregoing results concerning learning rate are robust to either choice rule. We adopt the sampling model because the process-level description of decision noise motivates analyses of neuroimaging data during choice formation, presented below.

At the fixed, slow learning rate, the best-fit value of 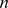 was 4.675+/−1.25 samples, across subjects. As in our learning rate analysis, we estimated this as a fixed effect (4.177), for generating our fMRI regressors (see *Choice difficulty* in *Neuroimaging results*).

### Neuroimaging

We next identified neural correlates of each learning process.

#### Stimulus anticipation in each process has distinct neural substrates

We began by looking for correlates of participants' anticipation of the next image to appear. Specifically, we sought activity that reflected how difficult it might be to predict this next image. Previous work [7], [9], [10] has shown that BOLD activity in hippocampus and elsewhere covaries with the participants' modeled uncertainty about future events. This may reflect a process of spreading activation, by which an image triggers activations of likely successor images, which are more numerous in situations of uncertainty. Also consistent with this idea, the anterior portion of the hippocampus was recently shown more directly to reflect such anticipation in sequential relationships among abstract stimuli [23].

Here, uncertainty is formally defined as the “forward entropy,” or entropy of the model's prediction about the identity of the next image, conditional on the current one. This is a trial-by-trial function of the model's learned transition probabilities, which in turn depend on the learning rate fit to behavior. These regressors are specified as parametric modulators on delta functions placed at the onset of the currently presented image.

**Figure 4:**
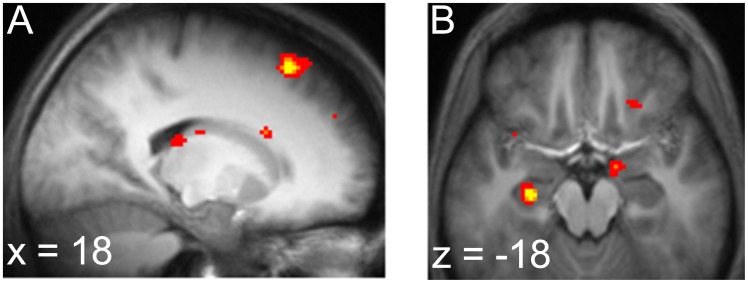
BOLD signal reflecting anticipation of the next stimulus. *a*. BOLD signal correlated with forward entropy in the fast process. Activity in the dorsal caudate was significant after correction over an anatomically-defined mask of bilateral caudate. *b*. BOLD signal correlated with forward entropy in the slow process. Activity in the anterior hippocampus was significant after correction over an anatomically-defined mask of left hippocampus. Both *a* and *b* displayed at 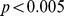, uncorrected.

The two-process model as fit to reaction times therefore gives rise to two entropy timeseries, one each from predictions generated at the fast and slow learning rates. Based on our previous results [7], we expected to find different correlates corresponding to the entropy timeseries from each process: in hippocampus for the slower learning rate and in striatum for the faster learning rate. We defined, using the AAL template library, anatomical masks of the structures in which we observed above-threshold activations in our previous study: left hippocampus for slow learning rate entropy and bilateral caudate for fast learning rate entropy [7]. Accordingly, when forward entropy was computed according to the slow learning rate process, a cluster of significantly correlated activity was observed in the region identified in our previous study, left anterior hippocampus (peak −26, −10, −18; 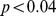 corrected for family-wise error due to multiple comparisons over an anatomically-defined mask of left hippocampus; Figure 4).

We ran a separate regression containing an identical GLM except for the entropy regressor, which was now computed according to the fast learning rate. In this GLM, we observed activation on the tail of right caudate (peak 24, −14, 26) that was significant when corrected for multiple comparisons over an anatomically-defined mask of bilateral caudate (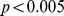). (A symmetric cluster in left caudate was observed at 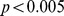 uncorrected, but did not survive correction for multiple comparisons.)

**Figure 5:**
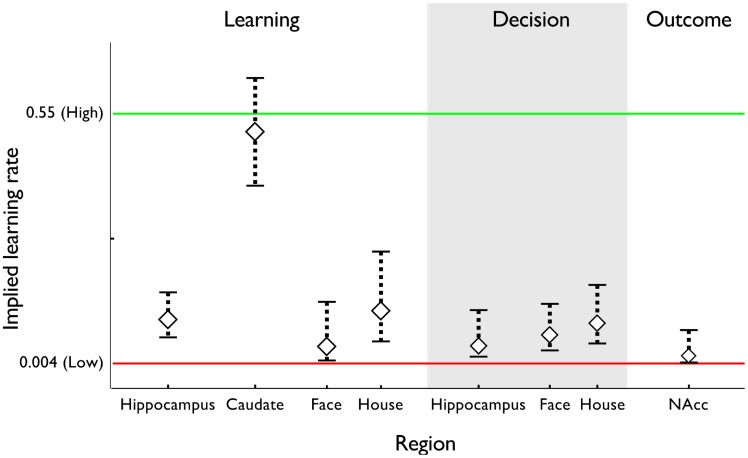
Learning rate 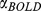 computed from BOLD signal. Learning rates computed from each of our regions of interest, overlaid on the learning rates fit to reaction time behavior. The best-fitting learning rates are displayed for each type of trial: sequential image-identification trials, decision trials, and choice outcome trials. For learning trials in hippocampus and caudate, learning rates are computed using the forward entropy regressor. For learning trials in face- and house-selective cortex, learning rates are computed using the estimated probability of the image appearing on the next trial. For decision trials in hippocampus, learning rate is computed using the choice difficulty regressor. For decision trials in face- and house-selective cortex, learning rates are computed using the portion of the choice difficulty regressor specific to that image. For outcome trials in nucleus accumbens, learning rate is computed using the reward prediction error regressor. Error bars: 1 SEM.

The foregoing results suggest two prediction processes that each learn at a rate corresponding to one of those observed in the RT behavior, with anatomically separate substrates. As in our previous study [7], we more directly tested the correspondence of learning rate to neural structure within a single GLM by independently estimating the learning rate that best explained entropy-related BOLD signals in each area. We located voxels of interest in an unbiased manner and fit the learning rate using a Taylor approximation to the entropy regressor's dependence on the parameter [7], [24], [25]. Neural learning rate estimates are visualized, superimposed over the behaviorally-obtained learning rates, in Figure 5.

Matching our previous results [7], the fast learning rate from RTs matched the one computed from BOLD signal in the striatum. In the mean over participants, the learning rate implied by BOLD in caudate was 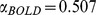. This rate was significantly larger than the slow learning rate fit to RTs (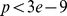), but not significantly different from the fast learning rate (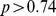).

In our prior study [7], the slow learning rate from RTs matched the one computed from BOLD signal in the anterior hippocampus; here, though the hippocampal BOLD learning rate (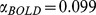) was numerically closer to the slow rate fit to RTs, it was statistically different from both that rate as well as the fast (both 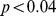). Importantly, however, it was not statistically distinguishable from the learning rate fit to choices (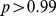) — thus supporting the critical link, from learning to choices — and also significantly smaller than the striatal learning rates computed from BOLD (paired samples; 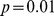).

Taken together with the behavioral model fits, these neuroimaging results and learning rate computations support the suggestion that two distinct processes learn to estimate the sequential contingencies embedded in our image identification task. Further, neural activity in two structures reflects anticipation (indexed by forward entropy) according to the estimates of each processes, with learning rates that differ from one another and approximate those identified in reaction time behavior.

#### Neural decision computations are uniquely explained by the slow process

We next sought correlates of decision computations driven by the learned transition probabilities. Our analysis of choice behavior indicated that decisions were informed by the sequential contingencies learned at a rate consistent with the slow learning rate fit to RTs. Therefore we hypothesized that activity related to decision computations would also be identified with a similar learning rate. If this indeed reflected a common underlying learning process, it would engage the anterior hippocampus, which was shown to support slow learning in the sequential learning task.

We first analyzed activity during the deliberation period leading up to the choice. Similar to our analysis of anticipatory activity during sequential response trials, we probed the neural correlates of deliberation by asking: how difficult was it for the participant to make this decision? We used as our measurement of choice difficulty the uncertainty (variance) in the decision variable (the value difference between options) that led to the current choice, computed using the choice model parameters fit to behavior (for details, see *Choice models* in *Materials and Methods*). This quantity, which was motivated by the process-level model of decision noise, is similar to the entropy measure used to define uncertainty during the learning task. The key difference is that the distribution being analyzed lumps images into two categories (rewarded vs nonrewarded) rather than predicting all four separately.

This regressor was specified at the time of onset of the choice screen.

**Figure 6:**
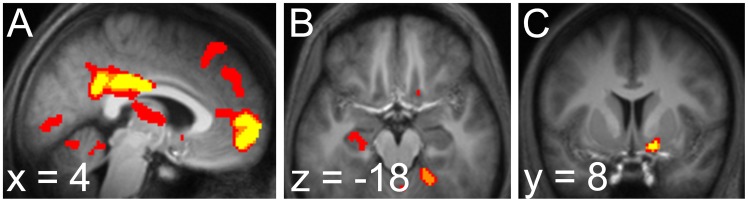
BOLD signal during choices and outcomes. During deliberation periods after choice options were presented, we observed activity in *a*. posterior cingulate (−2, −18, 32), anterior mPFC (4, 64, −2) and *b*. left hippocampus (peak −24, −10, −18), all significantly correlated with choice difficulty in the slow process. *c*. BOLD signal at outcome. A cluster in the nucleus accumbens (peak 10, 12, −2) correlated with reward prediction error as computed using the expectations derived from the slow process. All activations displayed at 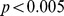, uncorrected.

In our region of prior interest, an area of left anterior hippocampus was activated, though only marginally significant after multiple comparison correction over our anatomical mask (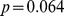; Figure 6b). This activation is similar to that seen to entropy during the stimulus prediction task.

Does this activity reflect learning similar to one of the processes observed in RT behavior? We again estimated the learning rate implied by these BOLD correlates. The learning rate computed from anterior hippocampal BOLD during choices matched the slow learning rate fit to RT. The mean learning rate that best explained this activity was 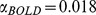 (Figure 5). This was different from the fast learning rate from RT behavior (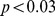), but did not differ from the slow RT learning rate (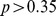). The involvement of the hippocampal region in both phases of the task, showing the same type of learned associations, supports the idea that a common learning process supports both behaviors.

#### Choice difficulty engages a fronto-temporal memory network

Additionally, at the whole brain level, the choice difficulty measure revealed correlates in a broad fronto-temporal network that appears to correspond to a component of the ‘default network’, a set of brain regions that has been associated with constructive memory and mindwandering [26], [27].

In particular, two clusters survived correction for multiple comparisons over the entire brain: a region of anterior medial PFC (peak 4, 64, −2; 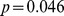), and a region of posterior cingulate cortex (peak −2, −18, 32; 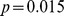; Figure 6a). Also, activation in a third component of the default network, the dorsomedial PFC (peak 14, 40, 40) survived whole-brain multiple comparison correction for cluster extent (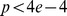), but not peak (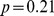). Together with the above-reported anterior hippocampal cluster, the overall pattern of activation is consistent with previous observations of the fronto-temporal memory component of the default network [28].

We ruled out alternative explanations for activity in these regions, or other variables that might correspond to the notion of ‘choice difficulty’. The choice difficulty regressor was not significantly correlated with reaction time (across subjects, mean 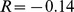), nor the expected value of the choice (mean 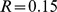).

#### Prediction error activity in striatum

This same hippocampally-linked, slow process learning also matched the neural reward prediction error (RPE) in nucleus accumbens [29]–[31]. We analyzed the RPE at the time of the onset of the first image following the choice, since that was the timepoint that primarily influenced the decision in our behavioral analysis. Here, the RPE is defined as the difference between the obtained reward (or $0, if an image other than the rewarded one occurs) and the expected value of the option chosen. Since the expected value depends on the learned image transition probabilities, this signal again should depend on the learning rate.

Figure 6 illustrates activity in nucleus accumbens correlated with the RPE regressor computed from the slow learning rate (peak 10, 12, −2 ; 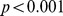 after correction for family-wise error due to multiple comparisons over an anatomical mask of the nucleus accumbens). Again, the learning rate in the NAcc was best matched to the slow learning rate fit to RT. The mean learning rate implied by NAcc activity was 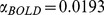. Across the population, this rate was smaller than the fast learning rate obtained from RT behavior (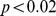) but was not different from the slow learning rate computed from RT behavior (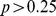). Thus, these results are again consistent with the idea that the choice phase of the task is driven by the slow, hippocampally-linked process.

To verify that these results are indiciative of a reward prediction error signal, and not simply driven by the receipt of reward, we extracted the coefficients for reward value and expectation separately. A signal reflecting the computation of reward prediction error should positively covary with the former, and negatively with the latter. This was in fact the case: across the population, the correlation coefficient at the peak voxel was significantly positive for reward value (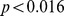, by two-tailed, one-sample t-test) and significantly negative for expected value (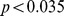).

#### Content-preferring visual regions are selectively driven by anticipation for stimulus category

One interpretation of activity related to forward entropy during the sequential image identification trials (Figure 4 above) is that it might result in the aggregate from the retrieval of likely targets in anticipation of the upcoming image. To seek more direct evidence for such retrieval at the item level, we leveraged the fact that our design used four category-specific exemplars as stimuli. Each of these exemplars was chosen because it represents a category that has been shown to preferentially engage a particular region of higher-order visual cortex: bodies [32], faces [33], houses [34], and household objects [35]. We examined whether activity in these regions was related to the estimated probability (from the model fit to participant behavior) that the corresponding image would appear on the *next* trial. This probability timeseries is a parametric measure of the strength of the estimate for a given image, specified at the time of onset of the preceding image. We tested these effects only for houses and faces, because these categories were the most consistently identified with regions in our initial localizer analysis.

**Figure 7:**
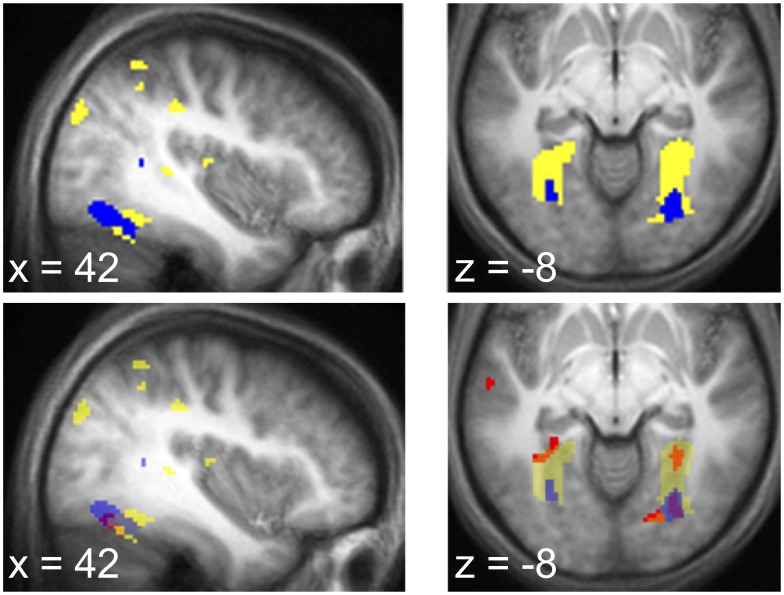
Image-selective regions. The regions defined by the in-task localizer contrasts house 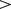 face and face 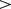 house, are colored yellow (left: face, right: house). The face localizer yielded the largest cluster of activation in a region of right fusiform gyrus. The house localizer yielded the largest cluster of activation in a region stretching from posterior parahippocampal gyrus to the occipital lobe. Regions selectively sensitive to the estimated probability of an image appearing next (on sequential response trials) are colored blue. Regions selectively sensitive to the difficulty of deciding whether a particular image would lead to reward are colored red. Displayed at 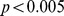, uncorrected.

First, we identified face- and house-sensitive regions using the relevant (in-task) localizer contrast: regions that responded more for trials on which the face was presented than they did on trials on which the house was presented, and vice-versa. We selected the voxels that survived correction over a combined anatomical mask of the right ventral stream regions: fusiform gyrus, parahippocampal gyrus, and inferior occipital lobe, chosen to encompass previously observed content-sensitive regions [32]–[35], and reflecting the fact that these activations tend to be right-lateralized in our areas of interest. The face and house selective regions are depicted in Figure 7 (face peak 42, −48, −20, 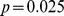 ; house peak 28, −82, −2, 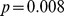).

These face- and house-selective regions were then used to seek activity sensitive in a graded fashion to *anticipation* of the face or the house, respectively. Within these regions, we tested for activity preferentially related to the probability of the face (as opposed to the house) appearing *next*, and vice versa. (Note that any such activity cannot be explained by a confounding tendency of the house actually to appear after it is expected, since the GLM also models the actual presentation of the faces and houses, and the test of the parametric effect of probability therefore turns only on the portion of activity orthogonal to this.) Indeed, activations within the face- and house-selective regions were significantly (though *negatively*) correlated with the probability of the corresponding image appearing next (face: peak 42, −66, −14, 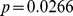; house: peak 26, −70, −8, 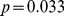). The face and house-selective regions and the corresponding contrasts selective for anticipation of each image are displayed in Figure 7.

Like entropy, the anticipatory probability regressor depends, in the model, on the learning rate that produces the probability estimates. We again estimated the learning rate, 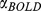, that best explained anticipatory activity in each of these category-selective regions (Figure 7). In both regions, the learning rate was best matched to the slow, hippocampal learning process. In the face region, the mean learning rate was 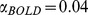. This rate was smaller than the fast learning rate fit to RTs (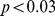), but not significantly different from the slow learning rate (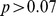). In the house region, the mean learning rate was 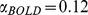. Across the population, this rate was numerically closer to the slow rate, but significantly different from both the fast and the slow However it did not significantly differ from other slow learning rates we estimated: that fit to choice behavior (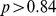), or the hippocampal learning rate computed from BOLD (paired samples; 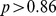). Finally, this rate *was* significantly smaller than the learning rate computed from striatal BOLD (paired samples; 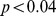).

Together, these results confirm that anticipatory activity in the image-sensitive regions corresponds with the estimated probability of each image appearing next. Further, they concord with the notion that learning implied by these signals most closely conforms to a slow learning process identified in reaction time, choice, and hippocampal BOLD.

#### Content-selective regions are selectively driven by difficulty of deliberating about a stimulus category

Activity in content-preferring regions was linked to the slow, hippocampal process during choice trials as well. Our choice model, fit to behavior, involved drawing samples of associations that would lead to the rewarded image. Here, we looked for activity in content-selective regions consistent with the reinstatement predicted by this process. For this analysis, we split our measure of choice difficulty into separate components, associated with each of the four different image categories (though limiting our analysis again to faces and houses). In particular, we considered the uncertainty about the probability that each image, separately, would lead to the rewarded image. We hypothesized that if the decision process involved retrieving each image's associates in attempting to compute its chance of leading to reward, then activity in the category-sensitive regions might be modulated by the difficulty of making this determination. Indeed, at the slow learning rate, the BOLD signal was positively correlated with the category-specific choice difficulty in the content-sensitive regions previously identified (face: peak 40, −62, −16, 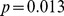; house: peak 30, −76, −6, 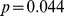 ; all p-values corrected for multiple comparisons over the respective regions identified in our visual localizer).

Again, the activity in both face and house-selective regions was best matched to the slow learning process. The mean learning rate implied by activity in the face-selective region was 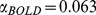. This rate was slower than the fast rate identified in RT behavior (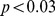), and did not differ significantly from the slow learning rate fit to RTs (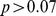). The mean learning rate implied by activity in the house-selective region was 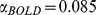. This rate was also smaller than the fast RT learning rate (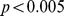). Consistent with our hypothesis, it did not differ significantly from the slow RT learning rate (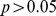).

For a full accounting of the comparisons between each of the learning rates identified in choices, reaction times, and BOLD, see Table 1.

**Table 1:** Learning rates implied by BOLD in each region of interest.

Region-Regressor	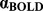	Not fast?	Not slow?	Not choice LR?	Not HC?	Not caudate?
HC-Entropy	0.099	*	* !	n.s.	-	*
HC-Difficulty	0.018	*	n.s.	n.s.	n.s.	*
Caudate-Entropy	0.507	n.s.	**	**	*	-
NAcc-RPE	0.0193	*	n.s.	n.s.	n.s.	*
Face-Probability	0.04	*	n.s.	n.s.	n.s.	*
Face-Difficulty	0.063	*	n.s.	n.s.	n.s.	*
House-Probability	0.12	*	* !	n.s.	n.s.	*
House-Difficulty	0.085	**	n.s.	n.s.	n.s.	**

* - 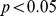.

** - 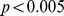.

! - test ran counter to our hypothesis about the learning rate of that region.

Taken together, these results tie activity in the ventral visual stream during decisions to an associative learning process consistent both anatomically and in terms of learning rate with that examined during sequential responding. Thus, altogether, these results suggest that the associative learning processes whose correlates were observed in hippocampus and the ventral visual stream during the sequential response trials also support deliberative, goal-directed planning in decisions for reward.

## Discussion

It is well established that decisions can be influenced by knowledge of contingencies embedded in the environment. The current study examined the neural computations underlying the learning of these contingencies, and linked them to computations underlying the decisions themselves. We present evidence that model-based decisions are supported by a contingency learning process involving hippocampus and ventral visual cortex, whose activity changed with this learning and was observed in concert with multiple kinds of instrumental behavior.

These results go beyond previous research that indirectly inferred the contribution of contingency learning to decisions, by using characteristics of the decisions and neural activity related to decision variables (action values or prediction errors; [36]–[38]), or conversely by examining activity related to contingency learning [15] without directly comparing it to choices. Here, we used additional observables — reaction times and fMRI signatures of reactivation of past experiences — to examine the learning of contingencies more directly, and to demonstrate that a component of this learning was quantitatively well matched to that implied by decisions.

### Learning rate comparisons

We are able to compare learning across different task phases (learning and choice) and sorts of measurements (reaction times, choices, and BOLD correlates of different quantities) by treating them all as different windows on a computational learning process. We fit each sort of data with a standard computational model of how predictions are learned from recent experience, and compare the learning rate parameters that best explain these measurements. The pattern of data in Figures 3 and 5 and Table 1 shows a striking consistency in these estimated learning rates between the different measurements.

However, there are a number of caveats to keep in mind about these analyses. First, it is in principle not possible to conclude that any two of these learning rate estimates are “the same” as one another — only that they are not statistically distingishable. But this pattern of negative findings is supported by positive ones, for instance that the differences between the various manifestations of “slow” and “fast” learning rates are significant (Table 1). Also, our findings that apart from exhibiting similar learning rates, neural activity during choice and decisions implicate common neural structures support the interpretation that all this activity relates to a common underlying learning process. Ultimately, however, establishing a definitive link between activity during learning and choice will require additional work using methods that can probe causal relationships between brain function and behavior.

A related point is that the estimates of learning rates from BOLD in Figure 5 consistently tend to be less extreme than their behavioral counterparts, i.e. slightly slower relative to the fast learning rate and faster relative to slow. In a couple of cases, this difference between BOLD and behavioral estimates is significant, seeming to contradict the interpretation that all these measurements reflect a common learning process. We believe this relates to another important set of caveats with this study, which is that it is methodologically challenging to estimate learning rates from BOLD data due to the nonlinear relationship between the learning rate and the decision variables that have BOLD correlates (entropy, etc.). To permit estimation, we approximate this relationship as linear using a first-order Taylor expansion [7], [25], [39]. This allows us to estimate the learning rate in the context of the same standard fMRI analysis (using a general linear model) as the rest of our results, and in turn means these analyses cope in the standard ways with the many methodological complications of fMRI (including for instance intersubject random effects, temporal and spatial autocorrelation, hemodynamics, and regressor colinearity). This method appears to perform robustly in this and our previous study [7] and other closely related analyses of parametric brain-behavior relationships [38], [40], [41], but there has not yet been a formal simulation study quantifying the error introduced by this approximation. One key sort of approximation error that we have examined [7] arises from our choice of the midpoint between fast and slow learning rates as the point around which to linearize. We choose this point to minimize the distance between the linearization point and the hypothetically relevant learning rates, since the error from linear extrapolation is expected to accumulate with distance. However, this choice interacts with the way we identify voxels of interest for fitting the learning rate, by identifying peaks in activity assuming this midpoint learning rate. Intuitively, this selection biases the estimated learning rates toward this midpoint (see our previous study using this approach for a more thorough technical explanation [7]). Although this effect is innocuous with respect to the conclusions in this article, it may account for some of the observed difference between neural and behavioral estimates in Figure 5.

### Hippocampus and striatum

Our choice task has one of the key features of a latent learning task [15]: sequential contingency learning precedes the introduction of a new and unpracticed rewarding goal. In particular, given the sparse occurrence of the choice probes, and the different combinations of rewarded and starting images, these decisions implicate a model-based response strategy requiring participants to evaluate options' chances of reaching the new goal based on the predictive associations being continually learned in the sequential image presentation trials. Conversely, choices of this sort leave little room for model-free reinforcement learning based only on the success of particular choices at earning money in previous choice trials.

Consistent with this, a key neural player in both the learning and decision phases in our results is the hippocampus. The hippocampal system is associated with flexible memory for stimulus-stimulus relations [42]–[44] and is a longstanding candidate for maintaining contingency structure in the service of goal-directed decisions [2], [19], [45]–[48]. In part, these suggestions are based on the analogy with spatial tasks, in which it has long been argued that the hippocampus implements a cognitive map [49], [50].

A suggestive connection of these ideas to nonspatial tasks is ubiquitous findings that the the hippocampal system is implicated in acquired equivalence, transitive inference, and sensory preconditioning effects [41], [51]–[53], as well as the flexible use of conceptual [54] and structured [55] knowledge. All of these effects demonstrate a bias in novel choice probes caused by previously learned stimulus-stimulus relations. Model-based decision making relies on a similar ability to flexibly chain together or recombine associations in novel ways, as exercised in latent learning tasks like our choice probes here.

Accordingly, we hypothesized that participants would draw on hippocampally-linked contingencies to make decisions. Indeed, the learning rates that best explained both choices and BOLD signals during the decision trials were not distinguishable from those seen in hippocampus and nearby ventral stream visual cortex during sequential responding, while differing significantly from those seen in BOLD activity in caudate and the fast process in reaction times. This quantitative convergence between learning processes examined during different tasks and through the lens of different observables substantiates the idea that model-based decisions and incidental stimulus-stimulus learning, like other sorts of relational learning and transfer [41], [53]–[55] are supported by the same hippocampal memory system.

Interestingly, the literature concerning these tasks suggests what appear to be two distinct (but potentially complementary) mechanisms supporting the flexible transfer of relational knowledge to novel probes. Some studies have demonstrated that better performance on transfer probes is predicted by hippocampal BOLD activity at learning but not test time [53], [56] suggesting that transfer is somehow supported by processes that occur already during encoding. One hypothesis is that such activity reflects the immediate transfer of learning, when information is first obtained, to other related associates by a process of spreading activation. In other studies [54], [55], neural activity at probe time also related to correct performance or with the relational information itself. This suggests the importance of processes occurring at the time of retrieval, and is consistent with theories (as in the standard account of model-based RL) that transfer is supported by some sort of active inference, planning or search at the time of the novel choice. Our result (discussed further below) that hippocampal activity tracked the difficulty of the decision probes speaks to the latter mechanism, providing relatively direct evidence that the hippocampal system engages in more computation for harder transfer problems (see also Simon & Daw [57]). Altogether, these two distinct but complementary mechanisms appear to be each well supported across the literature, and could plausibly both contribute in different circumstances.

The type of model-based decision making studied here contrasts with “model-free” habit learning, of the sort associated with dorsolateral striatum [58], predominant temporal-difference learning accounts of reward prediction error signal seen in dopamine neurons [6], and the striatal BOLD response [29]–[31]. That said, parts of striatum are clearly necessary for model-based decision making in rodents as well [59], [60]. Perhaps related, in human neuroimaging, even reward prediction errors observed in ventral striatum — though often characterized as reflecting the teaching signal for model-free stimulus-response learning — have recently been shown to report information about the state-state or relational structure of a task that would be known only to a model-based system [38], [41]. This may suggest some crosstalk between model-based and model-free learning in the brain. The reward prediction errors in the decision phase of the present task are consistent with these results, in that they reflect stimulus-stimulus predictions combined with trial-specific rewards to which a purely model-free reinforcement learner would be blind. The present results also extend these findings by showing that the stimulus-stimulus learning rate driving these prediction error effects matches that from the hippocampal system during the sequential response task, suggesting all these are indeed driven by a common learning process.

During the sequential response task, activity was *not* observed in the ventral striatal region commonly associated with reward prediction errors. This may reflect the lack of overt reinforcement in this more implicit association task. Instead, activity in a more dorsal/posterior region of striatum reflected a transient (high learning rate) adaptation process, which also had separate correlates in reaction times. We speculate that this activity (and the associated component of the reaction times) may reflect a second process of response learning, which did not carry over into the decision task. Indeed, the stimulus sequence in serial reaction time tasks of the sort we use is accompanied by an equivalent motor sequence (of button presses), leading previous authors to suggest [61]–[63] that participants might learn either or both of two distinct types of sequential associations: stimulus-stimulus and response-response. That these processes then are uniquely tied to separate brain systems — hippocampus and striatum — suggests that they reflect learning of information specialized to each of those systems. Given the broader functional roles of both structures, it is tempting to hypothesize that hippocampus is associated with stimulus-stimulus associations and striatum with response-response [64]–[66]. While we did not explicitly dissociate response-response and stimulus-stimulus associations, the weight of the literature tying each of these types of information to each brain structure suggests this hypothesis and encourages us to carry it forward throughout the below discussion. Importantly, by asking participants to seek a particular stimulus given another, our decision probes isolate only stimulus-stimulus associations and cannot be solved on the basis of response-response associations. Thus, the finding that the hippocampal activity (and its learning rate) contributed to these choices, but not the striatal one, is consisistent with these structures' hypothesized involvement in stimulus and response prediction. Further, the exclusive use of the slow-process associations in forward-looking, model-based choice suggest that these associations are of a type that may be flexibly recombined, a property long associated with hippocampal representations and not those of striatum [48], [52], [67].

That this learning was ‘slow’ in the hippocampus may at first seem to run counter to the notion that this structure supports flexible, rapidly bound learning, as in episodic memory. Model-based decisions are also characterized similarly, for instance because they tend to dominate behavior during initial learning but not following overtraining. However, it is important to emphasize that the theoretical ‘flexibility’ of the model-based system is in its ability to recombine the learned associations, applying them in novel contexts to novel goals: it is fundamentally about what is learned (e.g., a world model rather than a fixed policy) rather than how quickly. The question over what timescale any associations are learned is distinct from this issue – indeed, much previous work [57], [68] implies that the learning rate should normatively be controlled by factors such as the volatility of the environment and the reliability of observations. In this context, the learning rate measures the degree to which the model-based system can draw on experiences learned from the far past, in applying them to these novel contexts. A low learning rate indicates a long memory; a higher learning rate indicates a shorter memory.

The mechanisms which might give rise to these learning dynamics are an interesting topic for further research. Here, we have provided evidence that hippocampally-learned information is used in behavior via fetching memories of past transition events. That these candidate transition events might be drawn from memories stretching over tens of trials (spanning under a minute) into the past is well within understood capacity limitations of the hippocampal memory system. (For a further treatment of these issues, see the discussion provided in our previous paper using this task [7].)

### Anticipatory activation of stimulus representations

In category-selective regions of the ventral visual cortex, we observed reinstatement of stimulus-stimulus associations in a manner that was modulated by task demands, across our two different tasks. Over the sequential response trials, we observed that BOLD activity correlated with stimulus expectations in category-selective regions of the ventral visual stream. Specifically, activity in face- (or house-) selective regions of extrastriate visual cortex were also preferentially modulated by the expectation that the face (or house) image would appear next. The finding that activity parametrically fluctuates with stimulus predictions in both hippocampus and the ventral visual areas — and that the learning rates explaining these effects match one another — provides evidence that both areas are participating in a common associative learning process. At a more mechanistic level, it may be possible to interpret both entropy-related activity in hippocampus and probability-related activity in the ventral visual areas in terms of associative spreading that activates the representations of likely successors to the currently observed image.

On its face, the finding that anticipatory activity in the ventral areas *decreases* with conditional probability might seem to run counter to such a mechanism. That is, one might expect that, if probability is attributed largely to a single image, then the representation of that image should be more strongly activated. The contrary observation could be explained by a similar mechanism to the one that has been offered to explain ‘repetition suppression’ of BOLD (and spiking) responses [69], [70]. Here, a more narrowly tuned population could be recruited for more strongly expected stimuli. However, this explanation is insufficient to explain the parallel anticipatory activation we observe during choice trials, which are presumably the result of a common mechanism for anticipatory retrieval in the service of behavior.

A different interpretation of the effect is suggested by envisioning stimulus prediction as an active process of accessing memories. In particular, previously observed successors might be stochastically retrieved in a likelihood-weighted fashion to build up a statistical profile of the subsequent image, with this mnemonic evidence accumulated in a manner analogous to diffusion-to-bound models of perceptual discrimination [21], [71]. This idea is consistent with suggestions that anticipatory activity in category regions is driven by evidence accumulation [72]. If such a process terminates when evidence reaches some threshold, then spiking activity would be elevated only over a shorter interval of time and, thus, on trials with strong evidence observed signal would be lower when integrated over the length of the hemodynamic response [73].

The activity of these same category-selective regions during the decision trials could be understood in a similar manner, in terms of retrieving memories to evaluate candidate actions. Here, activity in the face (and house) areas of ventral visual cortex correlated with our measure of the *difficulty* of deciding whether the choice of that stimulus would lead to reward. This observation supports a model where evaluation of decision options occurs by bounded accumulation of evidence — memories stochastically sampled to evaluate the likely consequences of a choice (here, the successor image and its reward status).

### Episodic retrieval in forward search

Our aggregate (as opposed to stimulus-specific) choice difficulty measure was also positively correlated with activity in the anterior MPFC and posterior cingulate cortex. Activations under our reporting threshold were also observed in dorsal MPFC and anterior and posterior hippocampus. These regions together comprise the fronto-temporal memory component of the well-known “default network” [28]. Although originally characterized by its increased, coherent, activity during periods of rest, a role in deliberative evaluation is consistent with functional hypotheses for this network, in which activity is modulated by prospective or constructive memory. Tying together experimental data from multiple levels of observation and across task and rest modalities, Buckner & Carroll [26] suggest the default network “enables mental exploration of alternative perspectives based on our past experiences”, a proposal they expanded on in later discussions [27]. Burgess [74] offers a complementary suggestion for one component of the network, proposing that BA10 in particular acts as a ‘gateway’ between a focus on internal (e.g., mnemonic) and external (e.g., sensory) representations. These proposals — along with observations of hippocampus and default network activity during look-ahead planning [75]–[77] — concord with our interpretation of the choice difficulty correlate as reflecting reinstatement of prior experiences.

Finally, by offering a closer look at how the brain employs associations in the service of model-based decision making, our study suggests a route toward addressing one key puzzle in this area. To wit, whereas simple reward learning has a straightforward neural implementation (embodied in model-free temporal difference theories and relatives [6], [78], [79]), and the inference that these be accompanied by model-based choice is well established [3], the mechanism by which the brain actually implements such computations remains opaque. The idea we have advanced above, that successor states are retrieved stochastically (see also [45]), and their values integrated, connects directly with known neural mechanisms. In particular, although the idea of model-based planning as a mnemonic version of evidence accumulation differs at least superficially from more abstract conceptualizations based on tree search [3], [80], [81] or Bayesian inference [82], [83], sampling from successor states provides a more realizable process-level account of model-based evaluation in circumstances (such as chess) when the full set of future trajectories is too large to explore systematically. Moreover, it connects closely with evidence accumulation mechanisms that are well studied in the context of perceptual decision making, and comports with other suggestions that sampling or diffusion models apply to value-based decisions as well [28], [84]–[87]. It also joins those ideas with a literature suggesting that episodic memories can influence decisions [46], [56], [88].

## Materials and Methods

### Participants

Twenty-four right-handed individuals (twelve female; ages 18–40 years, mean 28) participated in the study. All had normal or corrected-to-normal vision. All participants received a fixed fee of $40 unrelated to performance, for their participation in the experiment, plus additional compensation of between $0 and $40 depending on their performance in one pseudorandomly-selected decision round. Participants were recruited from the New York University community as well as the surrounding area and gave informed consent in accordance with procedures approved by the New York University Committee on Activities Involving Human Subjects.

#### Exclusion criteria

Data from seven participants were excluded from analysis due to their being unusable for various reasons, leaving seventeen participants analyzed here. For three participants, this was due to failure to behaviorally demonstrate learning of the sequential contingencies embedded in the task. As we did in our previous study [7], we excluded subjects for failure to learn when a regression model with only nuisance regressors (the ‘constant’ model) proved a statistically superior explanation of participant RTs than any of the other models considered here, which each include regressors of interest specifying the estimated conditional probability of images (see *Analysis*, below). Statistical superiority over the constant model was measured by the Bayesian Information Criterion (BIC; [89]), used to correct likelihood scores when comparing models with different numbers of parameters. The rationale for excluding these subjects was that if they fail to learn the contingencies, it is not possible to ask the central question of the present study: how they use this learning to guide choices.

For the others, data were unusable due to operator error in operating the MRI unit (one participant), excessive head motion (two participants) and a failure to enter decisions on choice trials due to misunderstood instructions (one participant). Volumes during which instantaneous motion was 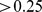 mm in any direction were excluded from analysis. Data from participants were excluded due to excessive motion when a large percentage (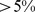) of volumes were excluded by this criterion.

### Task design

Participants performed a serial reaction time (SRT) task in which they observed a sequence of image presentations and were instructed to respond using a pre-trained keypress assigned to that image. The experiment was controlled by a script written in Matlab (Mathworks, Natick, MA, USA), using the Psychophysics Toolbox [90]. The stimulus set consisted of four grayscale images that were matched for size, contrast, and luminance. The images were chosen because they represent categories known to preferentially engage different areas of the ventral visual stream — bodies [32], faces [33], houses [34], and household objects [35]. Each participant viewed the same four images. During behavioral training, the keys corresponded to the innermost fingers on the home keys of a standard USA-layout keyboard (D, F, J, K). Participants were instructed to learn the responses as linking a finger and an image, rather than a key and an image (e.g. left index finger, rather than ‘F’). For the MRI sessions, the same fingers were used to respond on two MR-compatible button boxes. The mappings between the four images and four responses were one-to-one, pseudorandomly generated for each participant prior to their training session, trained to the criterion prior to the fMRI session, and fixed throughout the course of training and experiment sessions. Participants were informed that the key-to-image mapping was fixed, and that they were not being evaluated on the correctness of responses.

At each trial, one of the pictures was presented in the center of the screen, where it remained for three seconds, plus or minus uniformly distributed pseudorandom jitter, up to 474 ms in increments of 59 ms (the length of one slice in the MRI session). Participants were instructed to continue pressing keys until they responded correctly or ran out of time. Correct responses triggered a gray bounding box which appeared around the image for the lesser of 300 ms or the remaining trial time (Figure 1). Thus, each image presentation occurred for the programmed amount of time, regardless of participant response. The inter-trial interval consisted of 237 ms of blank screen.

The test phase of the scanning session proceeded with three blocks of 250 trials: 210 sequential response trials, 20 reward display screens (see *Choice trials*, below) and 20 choice trials. The first two blocks were followed by a rest period of participant-controlled length. During the rest period, participants were presented with a screen that was blank except for a fixation cross. Scan blocks after the first were initiated manually by the operator only after the participant pressed any of the relevant keys twice, to alert the operator that they were prepared to continue the task. Total experiment time — inclusive of training, practice and test periods — was approximately 1.5 hours, conducted continuously.

#### Stimulus sequence

For training, the sequence of images was selected according to a uniform distribution. Participants were instructed to emphasize learning the mappings between image and finger, disregarding speed of response in favor of correctly identifying the on-screen image.

In the test phase, participants were instructed to respond as quickly as they could, disfavoring accuracy as they had already been trained to criterion. The sequence of images was generated pseudorandomly according to a first-order Markov process, meaning that the probability of viewing a particular image was solely dependent on the identity of the previous image, with the conditional relationship specified by a 4×4 transition matrix (Figure 1). To motivate the choice trials, unlike in our previous study [7], participants were informed that conditional probability structure existed in the task. Four transition matrices were generated pseudorandomly at the start of the experiment for each subject, in a manner designed to balance two priorities: (i) to equalize the overall presentation frequencies for each image over the long and medium term (formally: fast mixing to a uniform stationary distribution), while (ii) examining response properties across a wide sample of conditional image transition probabilities. The procedure used to generate matrices satisfying these constraints is described in detail in our previous study [7].

Transition matrices were replaced at three evenly-spaced intervals — the second matrix was used starting on trial 188, the third matrix on trial 376, and the fourth on trial 563. Participants were informed that the structure would change, but they were not informed of when or how. The experiment display offered no indication of the shift to a different transition matrix, nor were matrix changes aligned with the onset of rest periods.

Time to first keypress was recorded as our primary behavioral dependent variable. Participants were not informed that RTs were being recorded, and no information was provided as to overall accuracy or speed either during or after the experiment. Trials on which the first keypress was incorrect were discarded from behavioral analysis.

#### Choice trials

Twenty choice rounds were interspersed throughout each of the three scanning sessions, for sixty choice rounds total per participant. Each choice round consisted of three parts (Figure 2). First, the reward display screen, visible for one second, notified the participant of which image was going to be rewarded and how much each occurrence of it would be worth. The rewarded image was chosen pseudorandomly from a uniform distribution over potential images. Reward values were whole dollar values between one and five, chosen pseudorandomly from a uniform distribution. Next, after a variable inter-stimulus interval of between two and eight seconds, chosen from a truncated exponential distribution with a mean of four, the participant was given five seconds to select between one of two *different* images. The two option images were chosen pseudorandomly from a uniform distribution, with the condition that they not be identical to the reward image. Participants were instructed to choose the image that was most likely to get them to the reward over the next few trials, and thereby earn the most money. Immediately after the choice was entered, the subsequent image was picked according to the conditional distribution implied by the image that the participant selected. The next image was then displayed after the standard ITI of 237 ms. Beginning with this first image after the choice — the ‘outcome’ image — text above each ensuing image indicated either a dollar amount (between $1 and $5), if it was the rewarded image, or $0 if it was not (Figure 2), for the extent of the choice round. The length of the choice round — that is, the number of images presented with dollar figures above them — was chosen from a truncated exponential distribution, with minimum of one, a maximum of eight and a mean of four, and adjusted to ensure a total of 80 trials across all of the choice rounds in a each session. To allow for equilibration of any transient effects, choice rounds did not occur within the first thirty trials of each scanning session.

### Analysis

Our analysis proceeded in several steps meant to first characterize the associative learning process, and then use this characterization to test behavioral and neural predictions about choices. Each participant's trial-by-trial RTs for correct identifications were regressed on explanatory variables including the estimated conditional probability of the picture currently being viewed given its predecessor — defined, in separate models (described below), in a number of different ways representing different accounts of learning — together with several effects of no interest. Trials on which the first keypress was not correct were excluded from behavioral analysis. Effects of no interest included stimulus-self transitions, image identity effects and a linear effect of trial number. Stimulus-self transitions were included to account for variance due to motor response readiness for the same keypress appearing twice in a row, above and beyond the preparation implied by any effect of the variables of interest. Image identity effects were included to account for any differential response time by each finger. Trial number effects were included to account for any monotonic shift in response time over the course of the experiment. These nuisance effects were identical across all models considered; the models differed in how they specified the explanatory variable of interest, the conditional probability of each image. In our initial analysis, the conditional probabilities were specified as the ground-truth contingencies: the probabilities actually encoded in the transition matrix. Having established that RT reflected such learning by demonstrating a significant correlation with these idealized probabilities (Figure 2), subsequent analyses used computational models to generate a timeseries of probability estimates such as would be produced by different learning rules with the same experience history as the participant (see *Learning models* for details). Similarly, the learning rules for conditional probability were fit (separately) to choices in the decision trials, estimated so as to maximize the likelihood that the model would have selected the same options as did the participant, given the same series of experience (see *Choice models* for details).

The learning models involved additional free parameters controlling the learning and decision processes (e.g. learning rates), which were jointly estimated together with the regression weights by maximum likelihood. For behavioral analysis, models were fit and parameters were estimated separately for each participant. At the group level, regression weights were tested for significance using a t-test on the individual estimates across participants [91]. To generate regressors for fMRI analysis (below) we refitted the behavioral model to estimate a single set of the parameters that optimized the RT and choice likelihoods aggregated over all participants (i.e. treating the behavioral parameters as fixed effects). This approach allowed us to characterize baseline learning-related activity separate from individual variation in neurally implied learning rates relative to this common baseline. For the former, in our experience [22], [25], [38], [92]–[95], enforcing common model parameters provides a simple regularization that improves the reliability of population-level neural results. Our neural model characterizes between-subjects variation in the learning rate parameter over this baseline, because it includes (as additional random effects across participants) the partial derivatives of each of the regressors of interest with respect to the learning rate.

#### Learning models

Based on our previous results analyzing contingency learning in an SRT task [7], we considered learning rules of the form proposed by Rescorla and Wagner [17] (see also [15]), which update entries in a 4×4 stimulus-stimulus transition matrix in light of each trial's experience. The appropriate estimate from this matrix at each step was then used as an explanatory variable for the RTs in place of the ground-truth probabilities.

Formally, at each trial the transition matrix was updated according to the following rule, for each image 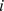:
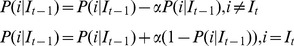
(1)where 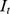 is the identity of the image observed at trial 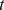 and 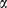 is a free learning-rate parameter. This rule preserves the normalization of the estimated conditional distribution.

Our primary model of interest for reaction times — again, drawn from our previous work [7] — was a weighted combination of two Rescorla-Wagner processes, each with different values of the learning rate parameter 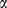.

Each process updated its matrix as above, independently, but the behaviorally expressed estimate of conditional probability was computed by combining the output of each process according to a weighted average with weight (a free parameter) 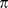:
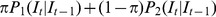
(2)


As the models considered here differ in the number of free parameters, we compared their fit to the reaction time data using Bayes factors ([96]; the ratio of posterior probabilities of the model given the data) to correct for the number of free parameters fit. We approximated the log Bayes factor using the difference between scores assigned to each model via the Laplace approximation to the model evidence [97]. This approximation was used because it provides a more fair comparison across models which use parameters of differing contributions to model complexity [98]. The evidence calculations assumed a uniform prior distribution for the values of the learning rate and weight parameters. In participants for whom the Laplace approximation was not estimable for any model (due to a non-positive definite value of the Hessian of the likelihood function with respect to parameters) the Bayesian Information Criterion [89] was instead used to estimate the posterior probabilities for all models. Model comparisons were computed both per individual, and on the log Bayes factors aggregated across the population.

#### Choice models

Each of the learning rates obtained from fitting reaction times also predicts a different series of option preferences on choice trials. We compared the relative fit to choice behavior of probability estimates at each learning rate or combination of learning rates. Each choice trial involves the choice between two options for the start image, which we index below as 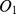 and 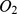, and a rewarded image, 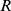.

We took as the decision variable the difference between the probability that each option would lead to the rewarded image in a single step: (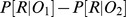), where the probabilities are the conditional image transition probabilities estimated by the learning model at the current point in the task. Motivated by race and sampling models [18], the model instantiates the decision variable on a particular trial by conducting some number 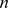 of draws from a binomial distribution around each learned transition probability. The mean proportion of successes on the first option is 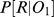, with binomial variance 
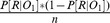
, and similarly for 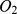. We estimate the choice likelihood by adopting a Gaussian approximation to the binomials, so that the resulting decision variable (the difference in sample proportions) has a mean and variance given by the difference and sum, respectively, of the means and variances of the two sample proportions. We compute the likelihood that the subject chooses 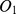 or 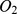 using the CDF of this Gaussian, and aggregate the log probabilities for the options actually chosen across the experiment to compute the likelihood of the choices given different probability learning models and parameters.

As fMRI regressors, we also use this model to define the per-trial choice difficulty as the variance of the decision variable (the sum of the binomial variances), and the per-category choice difficulty as the binomial variance of that category's probability estimate.

### fMRI methods

#### Acquisition

Imaging was performed on the 3T Siemens Allegra head-only scanner at the NYU Center for Brain Imaging, using a Nova Medical (Wakefield, MA, USA) NM011 head coil. For functional imaging, 40 T2*-weighted axial slices of 3 mm thickness and 3 mm in-plane resolution were acquired using a gradient-echo EPI sequence (TR = 2.37 seconds). Three scans of 400 acquisitions each were collected, with the first four volumes (9.48 seconds) discarded to allow for T1 equilibration effects. We also obtained a T1-weighted high-resolution anatomical image (MPRAGE, 1×1×1 mm) for normalization and localizing functional activations.

#### Imaging analysis

Preprocessing and data analysis were performed using Statistical Parametric Mapping software version 8 (SPM8; Wellcome Department of Imaging Neuroscience, London, UK). EPI images were realigned to the first volume to compensate for participant motion, co-registered to the anatomical image, and, to facilitate group analysis, spatially normalized to atlas space using a transformation estimated by warping the subject's anatomical image to match a template (SPM8 segment and normalize). Following the default settings in SPM, to account for warping due to normalization to the template image, data images were resampled to 2 mm (rather than 3 mm) isotropic voxels, in the normalized space [24]. Finally, data were smoothed using a 6-mm full-width at half maximum Gaussian filter. For statistical analysis, data were scaled to their global mean intensity and high-pass filtered with a cutoff period of 128 seconds. Volumes during which instantaneous motion was 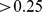 mm in any direction were excluded from analysis.

#### Statistical analysis

Statistical analyses of functional time-series were conducted using general linear models (GLM), and coefficient estimates from each individual were used to compute random-effects group statistics. Delta-function onsets were specified at the beginning of each stimulus presentation, and — to control for lateralization effects — nuisance onsets were specified for presentations on which right-handed responses were required. This had the effect of mean-correcting these trials separately. All further regressors were defined as parametric modulators over the initial, two-handed stimulus presentation or choice onsets. All regressors were convolved with SPM8's canonical hemodynamic response function. We used two separate GLMs for our main body of analyses: first, one analyzing sequential and response trials collectively, and a second breaking them down by image category.

In these GLMs we specify a number of parametric regressors derived from the model, often together with these regressors' partial dervatives with respect to the learning rate parameter. For the main analyses, all such regressors were evaluated using a (single) learning rate taken at the midpoint between the two identified in our best-fitting behavioral model, the two-learning rate model of Eqns 1 and 2. This enables us to detect activations related to these regressors without a bias toward one learning rate or the other, then use the partial derivatives to estimate the learning rate that best explains the signal (see *Learning rate analysis*).

We also performed ancillary GLM analyses to illustrate activations related to regressors computed using either learning rate identified in RT behavior. For these, the parametric regressors were substituted with the equivalent ones evaluated at one of those learning rates and the partial derivative regressors were omitted. Such analyses were carried out in separate GLMs due to correlation between regressors generated using different values of the learning rate parameter. However, it is important to note that these models were only used for generating figures to visualize the spatial extent of activity. Our formal results fitting learning rates to activity and comparing these estimates between areas are each conducted within a single GLM whose regressors (the main explanatory variable of interest and its partial derivative with respect to learning rate) in different weighted sums together approximately span the continuum of learning rates (see *Learning rate analysis*).. This allows the fit of different learning rates to an area to be formally assessed in a single model, while avoiding the problems of correlation between regressors and of specifying a discrete set of candidate learning rates a priori.

In all analyses, unless otherwise stated, activations are reported for areas where we had a prior anatomical hypothesis at a threshold of 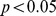 after correction for family-wise error (FWE) in a small volume defined by constructing an anatomical mask, comprising the regions of a priori interest. Our anatomical regions of a priori interest were: left hippocampus for slow process associations and bilateral caudate for fast process associations, based on our previous results [7]; right ventral stream cortical regions for visual localizer responses and anticipatory recall of category representations: fusiform gyrus, parahippocampal gyrus, and inferior occipital lobe, based on previous reports of visual category-selective patches of cortex — bodies [32], faces [33], houses [34], and household objects [35]; and nucleus accumbens, based on numerous previous reports of Reward Prediction Error (e.g. [30], [31], [38]). Anatomical regions were defined using the Automated Anatomical Labeling (AAL) atlas [99], except nucleus accumbens, which was taken from the mask produced in [38]. Masks were dilated by 4 mm in all directions to allow for inconsistencies in alignment with the population mean structural image. Unless otherwise stated, activations outside regions of prior interest are reported if they exceed a threshold of 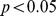, whole-brain corrected for family-wise error. All voxel locations are reported in MNI coordinates, and results are displayed overlaid on the average over participants' normalized anatomical scans.

#### GLM1: Main effects

The first GLM was used to analyze main effects of sequential response and choice trials. It contained the following regressors. First, to control for non-specific effects of reaction time (which, as demonstrated by our behavioral results, was correlated with our primary regressor of interest, the conditional probability), the RT on each sequential response trial was entered into the design matrix as a parametric nuisance effect. As a result all subsequent regressors, including all regressors of interest, were orthogonalized against this variable, ensuring that it accounted for any shared variance. We next included the conditional probability of the current image, to control for effects of surprise on the current trial. Building on our previous work [7], this regressor was not treated as a regressor of interest in our current experiment. Our primary regressor of interest on sequential response trials was the entropy of the distribution over the subsequent stimulus, given the image 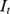 currently viewed:
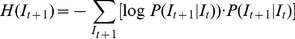
(3)where 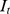 denotes the image displayed on trial t, but the sum is over all four possible image identities, 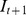. Whereas the conditional probability measures how ‘surprising’ is the current stimulus, this quantity, which we refer to as the ‘forward entropy’, measures the ‘expected surprise’ for the next stimulus conditional on the current one, i.e. the uniformity of the conditional probability distribution.

The entropy regressor was followed by the partial derivative of this forward entropy, with respect to the learning rate (see *Learning rate analysis*). Finally, nuisance regressors, last in orthogonalization priority, were entered to model variance due to the effects of: missed trials (those in which the participant did not press any keys in the allotted time), error trials, and self-transition trials (house-house, etc.).

For decision analysis, we specified onsets at the time of the presentation of the two options, and also at the first trial of the reward round, referred to as the ‘outcome’ trial. At the time options were presented, we first specified nuisance regressors: the reaction time of the choice, and the value of the rewarded image (between $1 and $5). Last were our primary regressors of interest: the difficulty of the choice (see *Choice models*), and the partial derivative of this regressor with respect to learning rate.

On outcome trials, we specified as a nuisance regressor the reaction time of the response. Following was our primary regressor of interest, the Reward Prediction Error (RPE): the reward received minus the expected value of the image chosen (the probability of receiving the reward image times the round's reward value), and its partial derivative with respect to learning rate.

#### GLM2: Image-specific effects

We used a second GLM to analyze image-specific effects in sequential response and choice trials. Critically, nuisance onsets were specified for trials on which each image category was presented. Additional nuisance onsets were specified for right handed choices and sequential responses, to control for effects of lateralization.

Onsets of interest were specified for sequential response and choice trials. For these analyses, we specified a set of four parametric regressors, one for each image type, over the sequential response and choice onsets. As we did not want our analysis to implicitly prioritize one or another variable, we disabled SPM's serial orthogonalization. On sequential response trials, our regressors of interest were the anticipated probability of each image — body, face, house, object — occuring next. We specified reaction time as a regressor of no interest, along with regressors for missed trials, errors, and self-self trials.

For choice trial onsets, we specified as the primary regressors of interest the choice difficulty for each category separately (see *Choice models*). Separate timeseries for the difficulty of deciding whether each image led to reward were modeled at every decision period (irrespective of whether that image was part of the decision set), and entered as parametric modulators over these onsets. Subsequent nuisance regressors were entered for the identity of the images on the screen, the identity of the rewarded image, the image categories used as options, the reward value, and the expected value of the decision. Again, these regressors were not orthogonalized against one another.

We also considered the possibility that analyses testing probability effects (Figure 7) were biased by selecting face- and house-sensitive voxels, then testing the effect of interest in those voxels in the same trials [100]. Accordingly, we measured the correlation between the selecting and testing regressors in the final design matrix. After filtering and whitening, the selecting and testing contrasts were not strongly correlated, and the mean of the measured correlation is in the opposite direction of the effect we observed (mean correlation coefficient across subjects: 0.1399+/−0.0238 for the face regressors, 0.0765+/−0.0308 for the house regressors). That is, to whatever extent there is a bias due to voxel selection, it would tend to work *against* the result we obtained.

#### Learning rate analysis

In the best-fitting behavioral model, the learned transition matrix arises from two modeled learning processes, each with a free parameter for its learning rate. Thus, a naive attempt to seek fMRI activations related to either hypothesized process separate from the other would need two separate but correlated sets of our various model-derived regressors of interest, such as entropy in sequential response trials and RPE on outcome trials. An alternative specification allows us to evade the problem of mutual correlation while also reasoning statistically about the learning rate that best explains BOLD activity related to a particular variable in a particular area.

To do this, we specify each regressor of interest in our GLMs together with its partial derivative with respect to the learning rate parameter. The weighted sum of these two regressors approximates (linearly, using a first-order Taylor expansion) how the modeled signal would change under different values of the learning rate parameter. Conversely, the best fitting learning rate can be approximated from the betas obtained for the two regressors [7], [25], [101]. Each regressor and its partial derivative were evaluated at the learning rate midway between the two behaviorally-obtained rate. The regression weight estimated for the derivative measures how far from the midpoint, and in which direction, was the learning rate that best explained BOLD. This analysis allowed us to formally investigate the possibility that learning rates expressed across regions of the brain (and multiple distinct computational variables) differed from one another, identify the pattern by which these learning rates varied, and compare them to the learning rates obtained from behavior.

Specifically, we constructed the regressors of interest as estimated by a single process learning at the rate 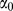 — which we set to the average of the two behaviorally identified rates — and included an additional regressor measuring how the 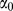 regressors would change if they had been generated from the model with a different learning rate. Technically, we defined these additional regressors as the partial derivatives of the original timeseries with respect to the learning rate parameter, evaluated at 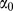 [101]. This analysis allows us to estimate the change in learning rate, relative to the reference point 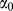, that would best explain BOLD in an area, by using a regression to estimate coefficients for the first two terms in the Taylor expansion of the dependence of the regressor on the learning rate. This takes the following form:
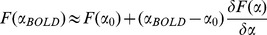
(4)


Here F(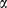) is the regressor of interest (i.e., the RPE or entropy timeseries), viewed as a function of the learning rate 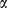, and 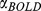 is some other learning rate for which the regressor would best fit the BOLD signal. To encode learning rates in this analysis, we used a change in variables by which the original Rescorla-Wagner learning rate was transformed by an inverse sigmoid, so that it ranged through the real numbers and estimates of it could be treated with Gaussian statistics. Thus, the learning rates reported from the fMRI response to the partial derivative (which includes a derivative of the sigmoid transform, by the chain rule), are sigmoid-transformed means of the underlying variable, 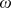. Similarly, the illustrated confidence bounds are the sigmoid-transformed S.E.M.s of 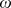.

This linear approximation to the (nonlinear) relationship between the regressor and the learning rate parameter allows the use of a GLM to approximately estimate the learning rates that would best explain BOLD correlates to the regressor. In particular, the weight estimated for the partial derivative regressor corresponds to 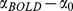 (or, more particularly, k[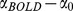], if the net effect of the regressor on BOLD is scaled by multiplying both sides of the approximation by some factor k). This is just the degree to which the best-fit (inverse-sigmoid transformed) learning rate for explaining the BOLD response differs from 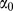, the value used to calculate our regressor of interest and its derivative.

We thus computed estimates of 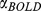 for each regressor (entropy or probability) at a voxel by first extracting the regression weights for the partial derivative regressor for each subject. To normalize these coefficients to a common scale in units of transformed learning rate (even if they originated from different regions), we divided these weights by the average, across subjects, of the regression weights for the corresponding regressor F(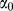) at the voxel, this corresponding to the overall scale factor k mentioned above. Lastly, we added the reference value 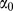, converting the result into the range of our behaviorally-obtained rates. Our statistical analyses were all performed on the learning rate estimates in the transformed units, taken across the population. Specifically, we test whether the computed 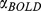 is statistically distinguishable from learning rate values obtained by fitting behavior, via t-tests against each (transformed) fit rate. We also test whether 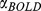 differs between regions, by comparing the estimates in paired-sample t-tests. For our plots of BOLD learning rates, we mapped the mean estimates and their confidence intervals through the sigmoid to depict them in units of Rescorla-Wagner learning rate.

To maximize power, to examine learning-rate effects at areas where there was learning-related activity, and to identify areas to allow between-region comparisons, we performed these analyses of leraning rates at voxels that we selected as peaks of contrasts on the main effect of the conditional probability, entropy, or prediction error regressors (not their derivatives), again using the midpoint rate 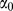. This was one motivation for choosing 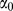 to be the midpoint of the fast and slow rates – i.e., that it is roughly equally suited to detect activity related to either rate. Additionally, the linear approximation to 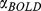 is most accurate when the difference 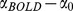 is small, suggesting a choice of 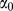 that is equally close to both relevant learning rates. We selected the voxels of peak group activation within each of our a priori regions of interest. Differences between parameters in the subsequent tests were considered reliable at a level of 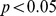.

Finally, note that selecting ROIs on the basis of correlation with a regressor of interest, then estimating the learning rate there, implies a bias that is innocuous with respect to our questions of interest, which generally concern to which of the extreme learning rates does the BOLD activity best correspond. It is intuitive — and can be shown [7] — that the estimated learning rate is biased toward the midpoint used for selection, and therefore away from the extremes that our hypothesis tests concern.

## Supporting Information

Figure S1Multiple views of the main effects. Saggital, coronal, and axial views of each of the effects reported in the main text. Each row displays activation corresponding to one of the parametric regressors: First, the forward entropy regressor, generated using the slow process. Second, the forward entropy regressor, generated using the fast process. Third, the choice difficulty regressor (views on the hippocampal correlates). Fourth, the choice difficulty regressor (views of the mPFC and PCC correlates). Fifth, the reward prediction error regressor. All images are displayed at a threshold of 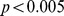, uncorrected.(TIFF)

Table S1Clusters greater than 10 contiguous voxels (at 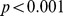) correlated with the forward entropy regressor computed at the slow learning rate.(TIFF)

Table S2Clusters greater than 10 contiguous voxels (at 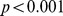) correlated with the forward entropy regressor computed at the fast learning rate.(TIFF)

Table S3Clusters greater than 10 contiguous voxels (at 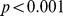) correlated with the choice difficulty regressor computed at the slow learning rate.(TIFF)

Table S4Clusters greater than 10 contiguous voxels (at 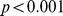) correlated with the reward prediction error regressor computed at the slow learning rate.(TIFF)

## References

[1] Dickinson A, Balleine BW (2002) The role of learning in the operation of motivational systems. In: Gallistel CR, Pashler HV, editors. Stevens Handbook of Experimental Psychology. Vol. 3: Learning, Motivation and Emotion. New York, NY: John Wiley & Sons Inc. pp. 497–533.

[2] Dickinson A (1980) Contemporary Animal Learning Theory. Cambridge: Cambridge University Press.

[3] Daw ND, Niv Y, Dayan P (2005) Uncertainty-based competition between prefrontal and dorsolateral striatal systems for behavioral control. Nature Neuroscience 8: 1704–1711. 16286932 10.1038/nn1560

[4] Thorndike EL (1911) Animal Intelligence. New York: Macmillan.

[5] Barto AC (1995) Adaptive Critics and the Basal Ganglia. In: Houk JC, Davis JL, Beiser DG, editors. Models of information processing in the basal ganglia, Cambridge, MA: MIT Press. pp. 215–232.

[6] Schultz W, Montague PR, Dayan P (1997) A Neural Substrate of Prediction and Reward. Science 275: 1593–1599. 9054347 10.1126/science.275.5306.1593

[7] Bornstein AM, Daw ND (2012) Dissociating hippocampal and striatal contributions to sequential prediction learning. European Journal of Neuroscience 35: 1011–1023. 22487032 10.1111/j.1460-9568.2011.07920.x PMC3325519

[8] Bahrick H (1954) Incidental learning under two incentive conditions. Journal of Experimental Psychology 47: 170–172. 13152289 10.1037/h0053619

[9] Strange BA, Duggins A, Penny W, Dolan RJ, Friston KJ (2005) Information theory, novelty and hippocampal responses: unpredicted or unpredictable? Neural Networks 18: 225–230. 15896570 10.1016/j.neunet.2004.12.004

[10] Harrison LM, Duggins A, Friston KJ (2006) Encoding uncertainty in the hippocampus. Neural Networks 19: 535–546. 16527453 10.1016/j.neunet.2005.11.002 PMC2640484

[11] Bestmann S, Harrison L, Blankenburg F, Mars R, Haggard P, et al (2008) Influence of contextual uncertainty and surprise on human corticospinal excitability during preparation for action. Current Biology 18: 775–80. 18485711 10.1016/j.cub.2008.04.051 PMC2387198

[12] Turk-Browne N, Scholl B, Johnson M, Chun M (2009) Neural evidence of statistical learning: efficient detection of visual regularities without awareness. Journal of Cognitive Neuroscience 21: 1934–45. 18823241 10.1162/jocn.2009.21131 PMC2773825

[13] Turk-Browne N, Scholl B, Johnson M, Chun M (2010) Implicit Perceptual Anticipation Triggered by Statistical Learning. Journal of Neuroscience 30: 11177–87. 20720125 10.1523/JNEUROSCI.0858-10.2010 PMC2947492

[14] Tolman EC (1948) Cognitive Maps in Rats and Men. Psychological Review 55: 189–208. 18870876 10.1037/h0061626

[15] Gläscher J, Daw N, Dayan P, O'Doherty JP (2010) States versus rewards: dissociable neural prediction error signals underlying model-based and model-free reinforcement learning. Neuron 66: 585–95. 20510862 10.1016/j.neuron.2010.04.016 PMC2895323

[16] Bush RR, Mosteller F (1956) A Stochastic Model with Applications to Learning. The Annals of Mathematical Statistics 24: 559–585.

[17] Rescorla RA, Wagner AR (1972) A Theory of Pavlovian Conditioning: Variations in the Effectiveness of Reinforcement and Nonreinforcement. In: Black AH, Prokasy WF, editors. Classical Conditioning II: Current research and theory. New York: Appleton-Century-Crofts. pp. 64–99.

[18] Ratcliff R (1978) A Theory of Memory Retrieval. Psychological Review 85: 59–108.

[19] Lengyel M, Dayan P (2008) Hippocampal Contributions to Control: The Third Way. Advances in Neural Information Processing Systems 20: 889–896.

[20] Erev I, Glozman I, Hertwig R (2008) What impacts the impact of rare events. Journal of Risk and Uncertainty 36: 153–177.

[21] Gold JI, Shadlen MN (2002) Banburismus and the Brain: Decoding the Relationship between Sensory Stimuli, Decisions, and Reward. Neuron 36: 299–308. 12383783 10.1016/s0896-6273(02)00971-6

[22] Daw ND, O'Doherty JP, Dayan P, Seymour B, Dolan RJ (2006) Cortical substrates for exploratory decisions in humans. Nature 441: 876–879. 16778890 10.1038/nature04766 PMC2635947

[23] Schapiro AC, Kustner LV, Turk-Browne NB (2012) Shaping of object representations in the human medial temporal lobe based on temporal regularities. Current Biology 22: 1622–7. 22885059 10.1016/j.cub.2012.06.056 PMC3443305

[24] Josephs O, Turner R, Friston K (1997) Event-Related fMRI. Human Brain Mapping 5: 243–248. 20408223 10.1002/(SICI)1097-0193(1997)5:4<243::AID-HBM7>3.0.CO;2-3

[25] Daw ND (2010) Trial-by-trial data analysis using computational models. In: Phelps E, Robbins T, Delgado M, editors. Affect, Learning and Decision Making, Attention and Performance. Xxiii edition. Oxford University Press.

[26] Buckner RL, Carroll DC (2006) Self-projection and the brain. Trends in Cognitive Sciences 11: 49–57. 17188554 10.1016/j.tics.2006.11.004

[27] Buckner RL, Andrews-Hanna JR, Schacter DL (2008) The brain's default network: anatomy, function, and relevance to disease. Annals of the New York Academy of Sciences 1124: 1–38. 18400922 10.1196/annals.1440.011

[28] Kahn I, Andrews-Hanna JR, Vincent JL, Snyder AZ, Buckner RL (2008) Distinct Cortical Anatomy Linked to Subregions of the Medial Temporal Lobe Revealed by Intrinsic Functional Connectivity. Journal of Neurophysiology 100: 129–139. 18385483 10.1152/jn.00077.2008 PMC2493488

[29] Delgado MR, Nystrom LE, Fissell C, Noll DC, Fiez JA (2000) Tracking the Hemodynamic Responses to Reward and Punishment in the Striatum. Journal of Neurophysiology 84: 3072–3077. 11110834 10.1152/jn.2000.84.6.3072

[30] O'Doherty JP, Dayan P, Friston K, Critchley H, Dolan RJ (2003) Temporal Difference Models and Reward-Related Learning in the Human Brain. Neuron 38: 329–337. 12718865 10.1016/s0896-6273(03)00169-7

[31] McClure SM, Berns GS, Montague PR (2003) Temporal prediction errors in a passive learning task activate human striatum. Neuron 38: 339–46. 12718866 10.1016/s0896-6273(03)00154-5

[32] Downing PE, Jiang Y, Shuman M, Kanwisher N (2001) A Cortical Area Selective for Visual Processing of the Human Body. Science 293: 2470–2473. 11577239 10.1126/science.1063414

[33] Kanwisher N, Mcdermott J, Chun MM (1997) The Fusiform Face Area: A Module in Human Extrastriate Cortex Specialized for Face Perception. Journal of Neuroscience 17: 4302–4311. 9151747 10.1523/JNEUROSCI.17-11-04302.1997 PMC6573547

[34] Epstein R, Kanwisher N (1998) A cortical representation of the local visual environment. Nature 392: 598–601. 9560155 10.1038/33402

[35] Malach R, Reppas JB, Benson RR, Kwong KK, Jlang H, et al (1995) Object-related activity revealed by functional magnetic resonance imaging in human occipital cortex. Proceedings of the National Academy of Sciences 92: 8135–8139. 10.1073/pnas.92.18.8135 PMC41110 7667258

[36] Hampton AN, Bossaerts P, O'Doherty JP (2006) The role of the ventromedial prefrontal cortex in abstract state-based inference during decision making in humans. Journal of Neuroscience 26: 8360. 16899731 10.1523/JNEUROSCI.1010-06.2006 PMC6673813

[37] Hampton AN, Bossaerts P, O'Doherty JP (2008) Neural correlates of mentalizing-related computations during strategic interactions in humans. Proceedings of the National Academy of Sciences 105: 6741–6746. 10.1073/pnas.0711099105 PMC2373314 18427116

[38] Daw ND, Gershman SJ, Seymour B, Dayan P, Raymond J (2011) Model-based influences on humans choices and striatal prediction errors. Neuron 69: 1204–1215. 21435563 10.1016/j.neuron.2011.02.027 PMC3077926

[39] Büchel C, Wise RJ, Mummery CJ, Poline JB, Friston KJ (1996) Nonlinear regression in parametric activation studies. NeuroImage 4: 60–6. 9345497 10.1006/nimg.1996.0029

[40] Wittmann BC, Daw ND, Seymour B, Dolan RJ (2008) Striatal activity underlies novelty-based choice in humans. Neuron 58: 967–73. 18579085 10.1016/j.neuron.2008.04.027 PMC2535823

[41] Wimmer GE, Daw ND, Shohamy D (2012) Generalization of value in reinforcement learning by humans. The European Journal of Neuroscience 35: 1092–104. 22487039 10.1111/j.1460-9568.2012.08017.x PMC3404618

[42] Squire LR (1992) Memory and the hippocampus: a synthesis from findings with rats, monkeys, and humans. Psychological Review 99: 195–231. 1594723 10.1037/0033-295x.99.2.195

[43] Cohen N, Eichenbaum H (1993) Amnesia, Memory and the Hippocampal System. Cambridge, MA: MIT Press.

[44] Rose M, Haider H, Salari N, Buchel C (2011) Functional Dissociation of Hippocampal Mechanism during Implicit Learning Based on the Domain of Associations. Journal of Neuroscience 31: 13739–13745. 21957237 10.1523/JNEUROSCI.3020-11.2011 PMC6633183

[45] Johnson A, Redish AD (2007) Neural ensembles in CA3 transiently encode paths forward of the animal at a decision point. Journal of Neuroscience 27: 12176–89. 17989284 10.1523/JNEUROSCI.3761-07.2007 PMC6673267

[46] Addis DR, Wong AT, Schacter DL (2007) Remembering the past and imagining the future: common and distinct neural substrates during event construction and elaboration. Neuropsychologia 45: 1363–77. 17126370 10.1016/j.neuropsychologia.2006.10.016 PMC1894691

[47] Daw ND, Shohamy D (2008) The Cognitive Neuroscience of Motivation and Learning. Social Cognition 26: 593–620.

[48] Buckner RL (2010) The role of the hippocampus in prediction and imagination. Annual Review of Psychology 61: 27–48, C1–8. 10.1146/annurev.psych.60.110707.163508 19958178

[49] O'Keefe J, Nadel L (1978) The hippocampus as cognitive map. Cambridge: Cambridge University Press.

[50] Redish AD (1999) Beyond the cognitive map: From place cells to episodic memory. Cambridge, MA: MIT Press.

[51] Bunsey M, Eichenbaum H (1996) Conservation of hippocampal memory function in rats and humans. Nature 379: 255–257. 8538790 10.1038/379255a0

[52] Dusek JA, Eichenbaum H (1997) The hippocampus and memory for orderly stimulus relations. Proceedings of the National Academy of Sciences 94: 7109–7114. 10.1073/pnas.94.13.7109 PMC21293 9192700

[53] Shohamy D, Wagner AD (2008) Integrating memories in the human brain: Hippocampal-midbrain encoding of overlapping event. Neuron 60: 378–89. 18957228 10.1016/j.neuron.2008.09.023 PMC2628634

[54] Kumaran D, Summerfield JJ, Hassabis D, Maguire EA (2009) Tracking the emergence of conceptual knowledge during human decision making. Neuron 63: 889–901. 19778516 10.1016/j.neuron.2009.07.030 PMC2791172

[55] Kumaran D, Melo HL, Duzel E (2012) The emergence and representation of knowledge about social and nonsocial hierarchies. Neuron 76: 653–66. 23141075 10.1016/j.neuron.2012.09.035 PMC3580285

[56] Wimmer G, Shohamy D (2012) Preference by association: How memory mechanisms in the hippocampus bias decisions. Science 338: 270–3. 23066083 10.1126/science.1223252

[57] Simon DA, Daw ND (2011) Environmental statistics and the trade-off between model-based and TD learning in humans. In: Shawe-Taylor J, Zemel RS, Bartlett P, Pereira F, Weinberger K, editors. Advances in Neural Information Processing Systems 24. pp. 127–135.

[58] Yin HH, Knowlton BJ, Balleine BW (2004) Lesions of dorsolateral striatum preserve outcome expectancy but disrupt habit formation in instrumental learning. European Journal of Neuroscience 19: 181–189. 14750976 10.1111/j.1460-9568.2004.03095.x

[59] Yin HH, Knowlton BJ (2006) The role of the basal ganglia in habit formation. Nature Reviews Neuroscience 7: 464–476. 16715055 10.1038/nrn1919

[60] Yin HH, Mulcare SP, Hilário MRF, Clouse E, Davis MI, et al (2009) Dynamic reorganization of striatal circuits during the acquisition and consolidation of a skill. Nature Neuroscience 12: 333–341. 19198605 10.1038/nn.2261 PMC2774785

[61] Davis DGS, Staddon JER (1990) Memory for reward in probabilistic choice: Markovian and non-Markovian properties. Behaviour 114: 37–64.

[62] Mayr U (1996) Spatial attention and implicit sequence learning: evidence for independent learning of spatial and nonspatial sequences. Journal of Experimental Psychology: Learning, Memory and Cognition 22: 350–364. 10.1037//0278-7393.22.2.350 8901340

[63] Willingham DB (1999) Implicit motor sequence learning is not purely perceptual. Memory & Cognition 27: 561–72. 10355244 10.3758/bf03211549

[64] Packard MG, White M, Ha Q (1989) Differential Effects of Fornix and Caudate Radial Maze Tasks: Evidence for Multiple Nucleus Lesions on Two Memory Systems. Journal of Neuroscience 9: 1465–1472. 2723738 10.1523/JNEUROSCI.09-05-01465.1989 PMC6569845

[65] McDonald RJ, White NM (1993) A triple dissociation of memory systems: hippocampus, amygdala, and dorsal striatum. Behavioral Neuroscience 107: 3–22. 8447956 10.1037//0735-7044.107.1.3

[66] Knowlton BJ, Mangels JA, Squire LR (1996) A neostriatal habit learning system in humans. Science 273: 1399–402. 8703077 10.1126/science.273.5280.1399

[67] Poldrack RA, Packard MG (2003) Competition among multiple memory systems: converging evidence from animal and human brain studies. Neuropsychologia 41: 245–51. 12457750 10.1016/s0028-3932(02)00157-4

[68] Behrens TEJ, Woolrich MW, Walton ME, Rushworth MFS (2007) Learning the value of information in an uncertain world. Nature Neuroscience 10: 1214–21. 17676057 10.1038/nn1954

[69] Li L, Miller EK (1993) The Representation of Stimulus Familiarity Temporal Cortex in Anterior Inferior. Journal of Neurophysiology 69: 1918–1929. 8350131 10.1152/jn.1993.69.6.1918

[70] Wiggs CL, Martin A (1998) Properties and mechanisms of perceptual priming. Current Opinion in Neurobiology 8: 227–33. 9635206 10.1016/s0959-4388(98)80144-x

[71] McClure SM, Gilzenrat MS, Cohen JD (2005) An exploration-exploitation model based on norepinephrine and dopamine activity. In: Advances in Neural Information Processing Systems. Cambridge, MA: MIT Press, pp. 867–874.

[72] Summerfield C, Trittschuh EH, Monti JM, Mesulam Mm, Egner T (2008) Neural repetition suppression reflects fulfilled perceptual expectations. Nature Neuroscience 11: 1004–1006. 19160497 10.1038/nn.2163 PMC2747248

[73] Philiastides M, Biele G, Heekeren H (2010) A mechanistic account of value computation in the human brain. Proceedings of the National Academy of Sciences 107: 9430–5. 10.1073/pnas.1001732107 PMC2889112 20439711

[74] Burgess PW, Dumontheil I, Gilbert SJ (2007) The gateway hypothesis of rostral prefrontal cortex (area 10) function. Trends in Cognitive Sciences 11: 290–8. 17548231 10.1016/j.tics.2007.05.004

[75] Schacter DL, Addis DR (2007) The cognitive neuroscience of constructive memory: remembering the past and imagining the future. Philosophical transactions of the Royal Society of London Series B, Biological sciences 362: 773–86. 17395575 10.1098/rstb.2007.2087 PMC2429996

[76] Viard A, Doeller CF, Hartley T, Bird CM, Burgess N (2011) Anterior hippocampus and goaldirected spatial decision making. Journal of Neuroscience 31: 4613–21. 21430161 10.1523/JNEUROSCI.4640-10.2011 PMC6622909

[77] Guitart-Masip M, Barnes GR, Horner A, Bauer M, Dolan RJ, et al (2013) Synchronization of medial temporal lobe and prefrontal rhythms in human decision making. The Journal of Neuroscience 33: 442–51. 23303925 10.1523/JNEUROSCI.2573-12.2013 PMC3562870

[78] Houk J, Adams J, Barto A (1995) A model of how the basal ganglia generate and use neural signals that predict reinforcement. In: Houk JC, Davis JL, Beiser DG, editors. Models of information processing in the Basal Ganglia. Cambridge, MA: MIT Press. pp. 249–270.

[79] Frank MJ, Seeberger LC, O'Reilly RC (2004) By carrot or by stick: cognitive reinforcement learning in Parkinsonism. Science 306: 1940–1943. 15528409 10.1126/science.1102941

[80] Keramati M, Dezfouli A, Piray P (2011) Speed/Accuracy Trade-Off between the Habitual and the Goal-Directed Processes. PLoS Computational Biology 7: e1002055. 21637741 10.1371/journal.pcbi.1002055 PMC3102758

[81] Wunderlich K, Dayan P, Dolan RJ (2012) Mapping value based planning and extensively trained choice in the human brain. Nature Neuroscience 15: 786–91. 22406551 10.1038/nn.3068 PMC3378641

[82] Botvinick M, An J (2008) Goal-directed decision making in prefrontal cortex: A computational framework. In: Koller D, Bengio, Y, Schuurmans D, Bouttou L, Culotta A, editors. Advances in Neural Information Processing Systems. Volume 21. pp. 169–176. PMC4171955 25258502

[83] Solway A, Botvinick MM (2012) Goal-directed decision making as probabilistic inference: A computational framework and potential neural correlates. Psychological Review 119: 120–54. 22229491 10.1037/a0026435 PMC3767755

[84] Rangel A, Camerer C, Montague PR (2008) A framework for studying the neurobiology of valuebased decision making. Nature Reviews Neuroscience 9: 545–56. 18545266 10.1038/nrn2357 PMC4332708

[85] Krajbich I, Rangel A (2011) Multialternative drift-diffusion model predicts the relationship between visual fixations and choice in value-based decisions. Proceedings of the National Academy of Sciences 108: 13852–13857. 10.1073/pnas.1101328108 PMC3158210 21808009

[86] Stewart N, Chater N, Brown GDA (2006) Decision by sampling. Cognitive Psychology 53: 1–26. 16438947 10.1016/j.cogpsych.2005.10.003

[87] Bornstein AM, Daw ND (2011) Multiplicity of control in the basal ganglia: computational roles of striatal subregions. Current Opinion in Neurobiology 21: 374–80. 21429734 10.1016/j.conb.2011.02.009 PMC3269306

[88] Peters J, Büchel C (2010) Episodic future thinking reduces reward delay discounting through an enhancement of prefrontal-mediotemporal interactions. Neuron 66: 138–48. 20399735 10.1016/j.neuron.2010.03.026

[89] Schwarz G (1978) Estimating the Dimension of a Model. Annals of Statistics 6: 461–464.

[90] Brainard DH (1997) The Psychophysics Toolbox. Spatial Vision 10: 433–6. 9176952

[91] Holmes AP, Friston KJ (1998) Generalisability, Random Effects & Population Inference. Neuroimage 7: S754.

[92] Simon DA, Daw ND (2011) Neural Correlates of Forward Planning in a Spatial Decision Task in Humans. Journal of Neuroscience 31: 5526–5539. 21471389 10.1523/JNEUROSCI.4647-10.2011 PMC3108440

[93] Schönberg T, Daw ND, Joel D, O'Doherty JP (2007) Reinforcement learning signals in the human striatum distinguish learners from nonlearners during reward-based decision making. Journal of Neuroscience 27: 12860–7. 18032658 10.1523/JNEUROSCI.2496-07.2007 PMC6673291

[94] Schönberg T, O'Doherty JP, Joel D, Inzelberg R, Segev Y, et al (2010) Selective impairment of prediction error signaling in human dorsolateral but not ventral striatum in Parkinson's disease patients: evidence from a model-based fMRI study. NeuroImage 49: 772–81. 19682583 10.1016/j.neuroimage.2009.08.011

[95] Gershman SJ, Pesaran B, Daw ND (2009) Human reinforcement learning subdivides structured action spaces by learning effector-specific values. Journal of Neuroscience 29: 13524–31. 19864565 10.1523/JNEUROSCI.2469-09.2009 PMC2796632

[96] Kass RE, Raftery AE (1995) Bayes Factors. Journal of the American Statistical Association 90: 773–795.

[97] Mackay DJC (2003) Information Theory, Inference, and Learning Algorithms. Cambridge, UK: Cambridge University Press. doi:10.2277/0521642981.

[98] Boone EL, Ye K, Smith EP (2005) Assessment of two approximation methods for computing posterior model probabilities. Computational Statistics & Data Analysis 48: 221–234.

[99] Tzourio-Mazoyer N, Landeau B, Papathanassiou D, Crivello F, Etard O, et al (2002) Automated anatomical labeling of activations in SPM using a macroscopic anatomical parcellation of the MNI MRI single-subject brain. NeuroImage 15: 273–89. 11771995 10.1006/nimg.2001.0978

[100] Kriegeskorte N, Simmons WK, Bellgowan PSF, Baker CI (2009) Circular analysis in systems neuroscience: the dangers of double dipping. Nature Neuroscience 12: 535–40. 19396166 10.1038/nn.2303 PMC2841687

[101] Friston KJ, Josephs O, Rees G, Turner R (1997) Nonlinear Event-Related Responses in fMRI. Magnetic Resonance Methods 39: 41–52. 10.1002/mrm.1910390109 9438436

