# Supplementary figures and images for: Cortical and Hippocampal Correlates of Deliberation during Model-Based Decisions for Rewards in Humans
Source: PLoS Comput Biol. 2013 Dec 5;9(12):e1003387. doi: 10.1371/journal.pcbi.1003387 (PMC3854511; doi:10.1371/journal.pcbi.1003387)

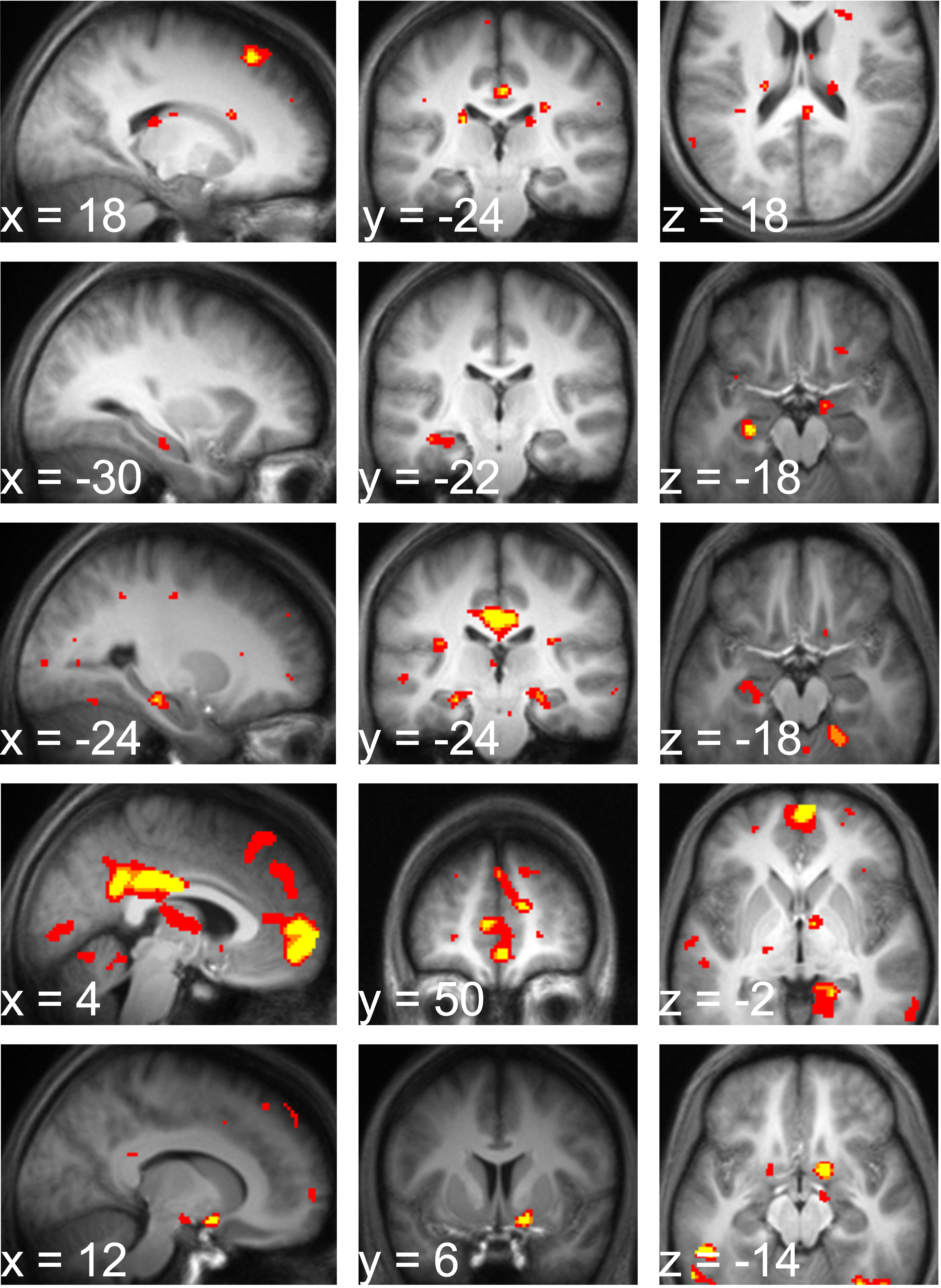

Supplement: Figure S1 — Multiple views of the main effects. Saggital, coronal, and axial views of each of the effects reported in the main text. Each row displays activation corresponding to one of the parametric regressors: First, the forward entropy regressor, generated using the slow process. Second, the forward entropy regressor, generated using the fast process. Third, the choice difficulty regressor (views on the hippocampal correlates). Fourth, the choice difficulty regressor (views of the mPFC and PCC correlates). Fifth, the reward prediction error regressor. All images are displayed at a threshold of , uncorrected. (TIFF) [file pcbi.1003387.s001.tiff]

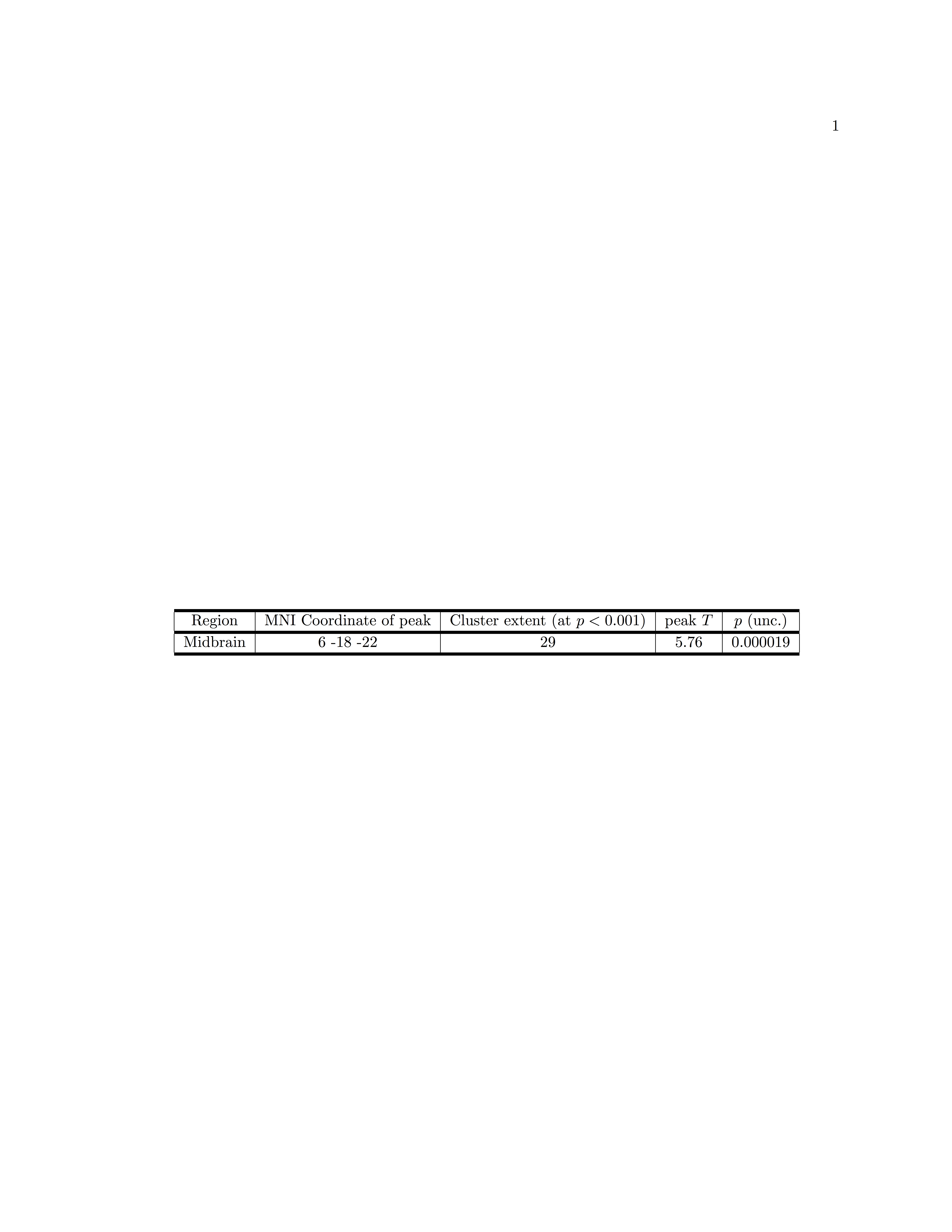

Supplement: Table S1 — Clusters greater than 10 contiguous voxels (at ) correlated with the forward entropy regressor computed at the slow learning rate. (TIFF) [file pcbi.1003387.s002.tiff]

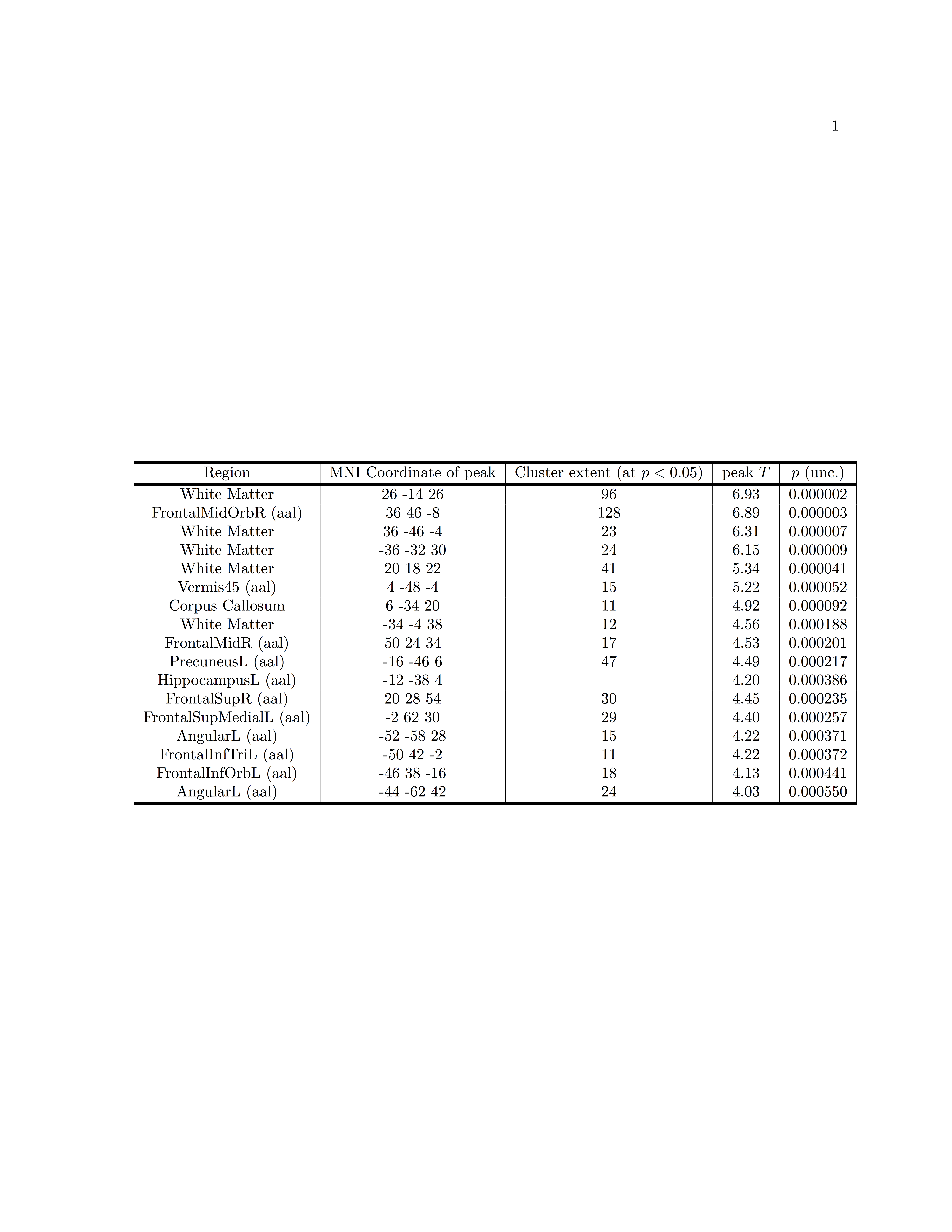

Supplement: Table S2 — Clusters greater than 10 contiguous voxels (at ) correlated with the forward entropy regressor computed at the fast learning rate. (TIFF) [file pcbi.1003387.s003.tiff]

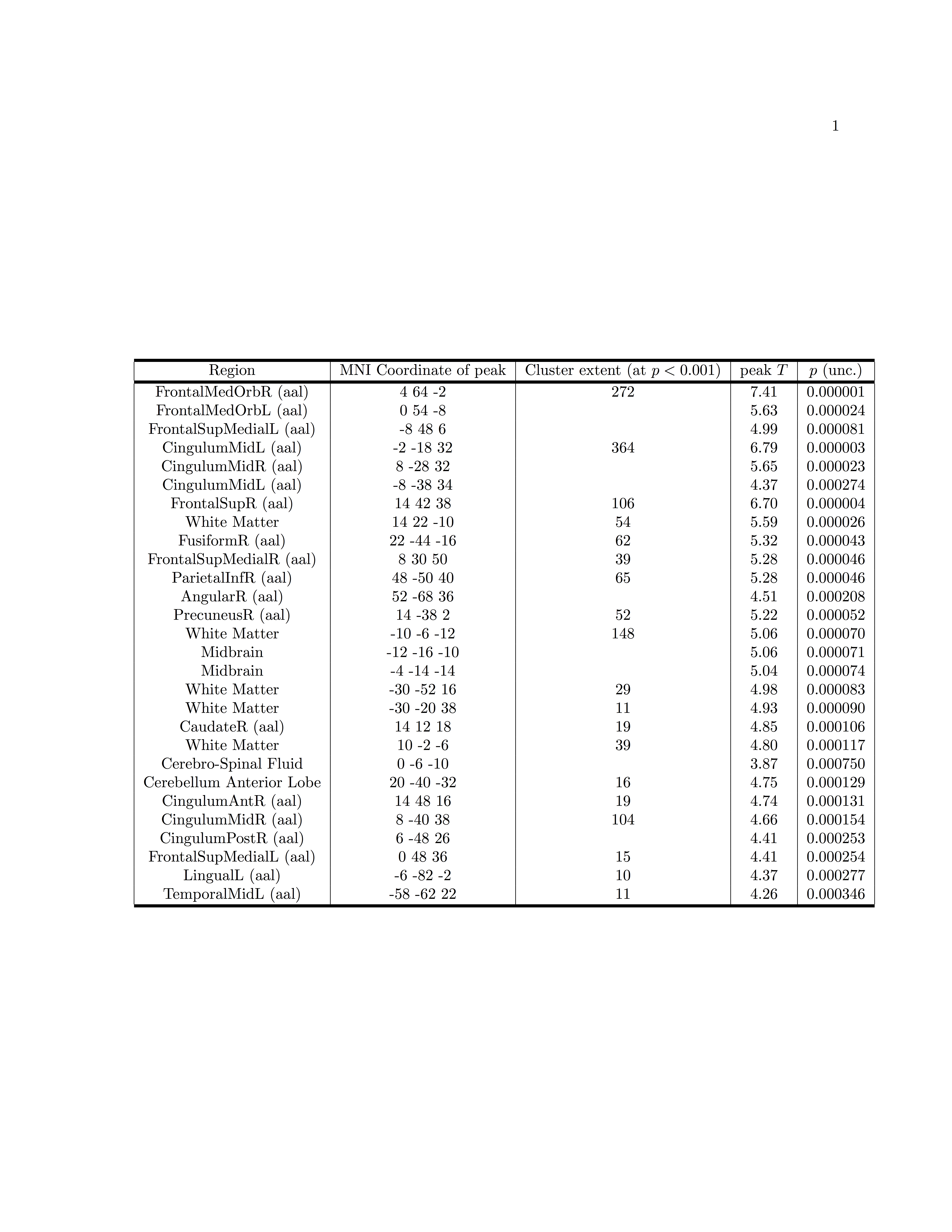

Supplement: Table S3 — Clusters greater than 10 contiguous voxels (at ) correlated with the choice difficulty regressor computed at the slow learning rate. (TIFF) [file pcbi.1003387.s004.tiff]

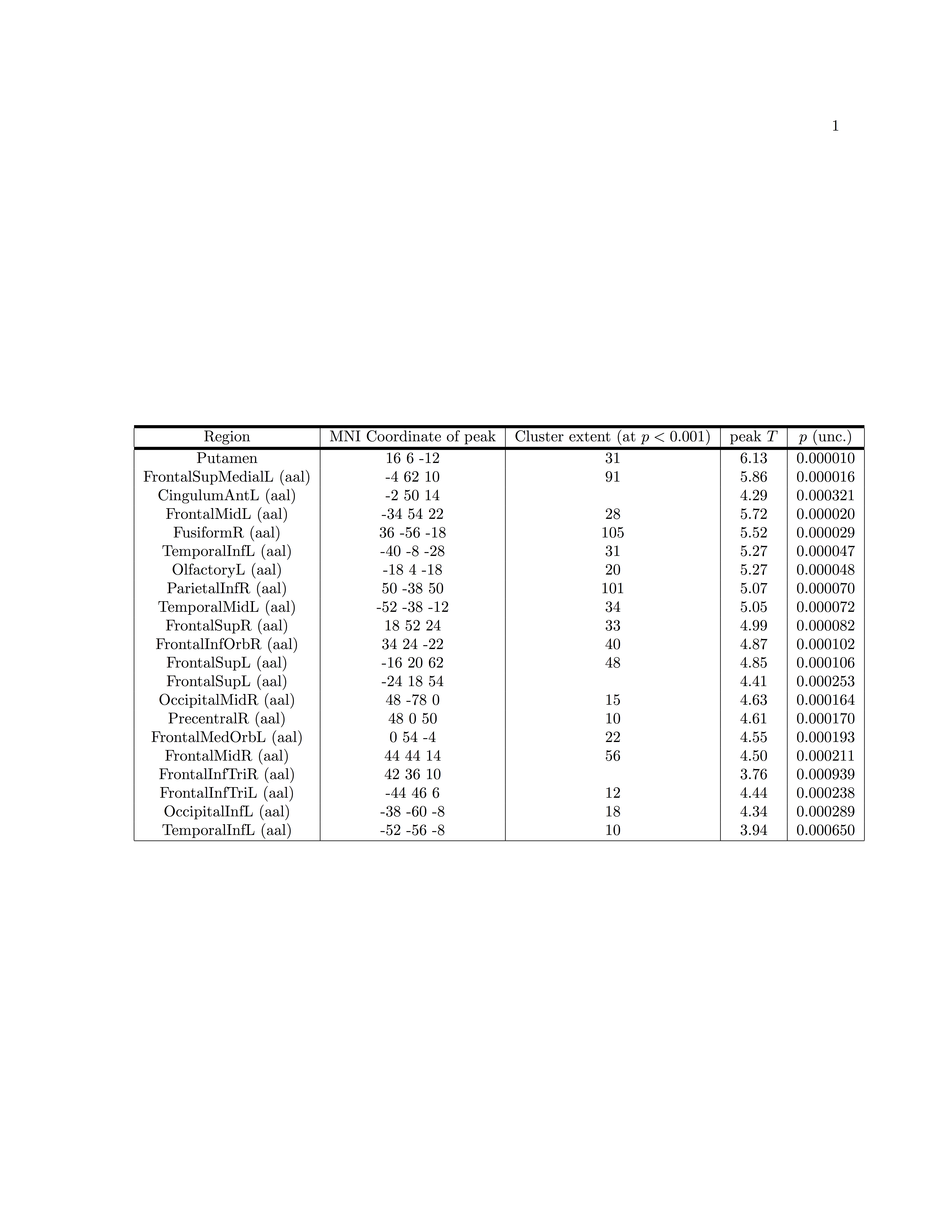

Supplement: Table S4 — Clusters greater than 10 contiguous voxels (at ) correlated with the reward prediction error regressor computed at the slow learning rate. (TIFF) [file pcbi.1003387.s005.tiff]
